# Hijacking of the host cell Golgi by *Plasmodium berghei* liver stage parasites

**DOI:** 10.1242/jcs.252213

**Published:** 2021-05-20

**Authors:** Mariana De Niz, Reto Caldelari, Gesine Kaiser, Benoit Zuber, Won Do Heo, Volker T. Heussler, Carolina Agop-Nersesian

**Affiliations:** 1 Institute of Cell Biology, University of Bern, CH-3012 Bern, Switzerland; 2 Institute for Anatomy, University of Bern, CH-3012 Bern, Switzerland; 3 Dept. of Biological Sciences, Korea Advanced Institute of Science and Technology (KAIST), Daejeon 305-701, Republic of Korea

**Keywords:** Host-parasite interaction, Host cell Golgi, CLASP, Golgi-associated vesicular traffic, *Plasmodium* liver-stages, Organelle hijacking

## Abstract

The intracellular lifestyle represents a challenge for the rapidly proliferating liver stage *Plasmodium* parasite. In order to scavenge host resources, *Plasmodium* has evolved the ability to target and manipulate host cell organelles. Using dynamic fluorescence-based imaging, we here show an interplay between the pre-erythrocytic stages of *Plasmodium berghei* and the host cell Golgi during liver stage development. Liver stage schizonts fragment the host cell Golgi into miniaturized stacks, which increases surface interactions with the parasitophorous vacuolar membrane of the parasite. Expression of specific dominant-negative Arf1 and Rab GTPases, which interfere with the host cell Golgi-linked vesicular machinery, results in developmental delay and diminished survival of liver stage parasites. Moreover, functional Rab11a is critical for the ability of the parasites to induce Golgi fragmentation. Altogether, we demonstrate that the structural integrity of the host cell Golgi and Golgi-associated vesicular traffic is important for optimal pre-erythrocytic development of *P. berghei*. The parasite hijacks the Golgi structure of the hepatocyte to optimize its own intracellular development.

This article has an associated First Person interview with the first author of the paper.

## Abstract (precis)

**Summary:** Recruitment of the host cell Golgi by the parasitophorous vacuolar membrane of *Plasmodium berghei* is crucial for optimal pre-erythrocytic development. The interaction is accompanied by parasite-induced formation of miniaturized host cell Golgi.

## INTRODUCTION

Malaria is a mosquito-borne infectious disease caused by parasites of the genus *Plasmodium.* In the mammalian host, the infection of the liver represents a bottleneck in the life cycle of the parasite. Hepatocyte infections are clinically silent, nevertheless, development of the parasite within the liver is extremely efficient, reportedly, one of the fastest growth rates among eukaryotic organisms (Prudencio et al., 2006). To accomplish its extensive replication, the parasite depends not only on *de novo* synthesis of essential metabolites, but also on scavenging and storage of host-derived nutrients (Bano et al., 2007; Inácio et al., 2015; Itoe et al., 2014; Meis et al., 1984; Niklaus et al., 2019; Petersen et al., 2017; Sá e Cunha et al., 2017; Zuzarte-Luís et al., 2017).

A critical factor for a productive intracellular replication of *Plasmodium* is the ability to successfully form a parasitophorous vacuole (PV). The parasitophorous vacuolar membrane (PVM) is the interface between the host cell cytosol, and the developing parasite. Although the role of the PVM and its molecular composition in liver stages is not fully understood, this structure is thought to play a key role in nutrient acquisition (Bano et al., 2007; Fougère et al., 2016; Grützke et al., 2014; Petersen et al., 2017), waste disposal and protection of the parasite from the host cell intracellular defense system (Agop-Nersesian et al., 2017; Boonhok et al., 2016; Grüring et al., 2012; Prado et al., 2015; Real et al., 2018; Spielmann et al., 2012). *Plasmodium*, like other intravacuolar pathogens, is believed to have evolved multiple strategies to access host cell resources. Key elements include the generation of an export machinery, modification of PVM permeability to enable the uptake of cytosolic metabolites, and subversion of host cell organelles (Agop-Nersesian et al., 2018; Ingmundson et al., 2014; Spielmann et al., 2012).

The Golgi is a key member of the secretory pathway and structurally consists of multiple disc-like cisternae that form stacks with *cis*-to-*trans* polarity. Proteins received from the endoplasmic reticulum (ER) at the *cis*-Golgi are further processed and sorted for transport at the *trans-*Golgi network (TGN) from which they are destined to other intra- or extracellular locations. Aside from protein distribution, both the Golgi and the ER are major sites of lipid synthesis and glycosylation. Distinct processing, sorting and synthesis events take place in an ordered sequence within discrete regions of the Golgi complex (Allan et al., 2002; Brandizzi and Barlowe, 2013; De Matteis and Luini, 2008; Wilson et al., 2011). Compromise of the Golgi structure alters the overall function of this organelle. Several proteins are involved in the maintenance of the Golgi architecture, including members of the proteinaceous scaffold known as the Golgi matrix, coat protein complex I (COPI), SNAREs and small GTPases, including multiple members of the Rab protein family (Fig. S1, Table S1). Various pathogens, including viruses, bacteria, and parasites, have been shown to interact with some of these proteins to scavenge nutrients and/or functionally or structurally alter the host cell Golgi (hcGolgi) (Deffieu et al., 2019; Heuer et al., 2009; Mañes et al., 2003; McGovern et al., 2018; Romano et al., 2017, 2013; Roy et al., 2006; Spanò and Galán, 2018; Spearman, 2018; Spriggs et al., 2019; Stein et al., 2012; Weber and Faris, 2018).

While PVM composition and nutrient incorporation is a topic of extensive research, *Plasmodium*-mediated subversion of host organelles remains understudied. Bano et al. have shown that *Plasmodium berghei* pre-erythrocytic stages settle at the juxtanuclear region of the host cell, and that throughout the development of the parasite, the PVM becomes associated with the host cell ER. While hcGolgi fragmentation has been described (Bano et al., 2007), in-depth investigation of the relationship of this organelle with *Plasmodium* was not undertaken.

In the present work, we explored the dynamics of interactions between the *P. berghei* PVM and the hcGolgi complex. We observed that pre-erythrocytic stages recruit the hcGolgi to the PVM, which acts as an interface between the parasite and the host. Parasites with an impaired PVM function displayed deficiencies in hcGolgi recruitment, and disruption of hcGolgi structural integrity led to restricted parasite growth. Altogether, we determined that recruitment of and sustained association with the hcGolgi is key for the survival and development of the parasite.

## RESULTS

### Recruitment of the host cell Golgi to the *P. berghei* PVM

**Fig. 1.**
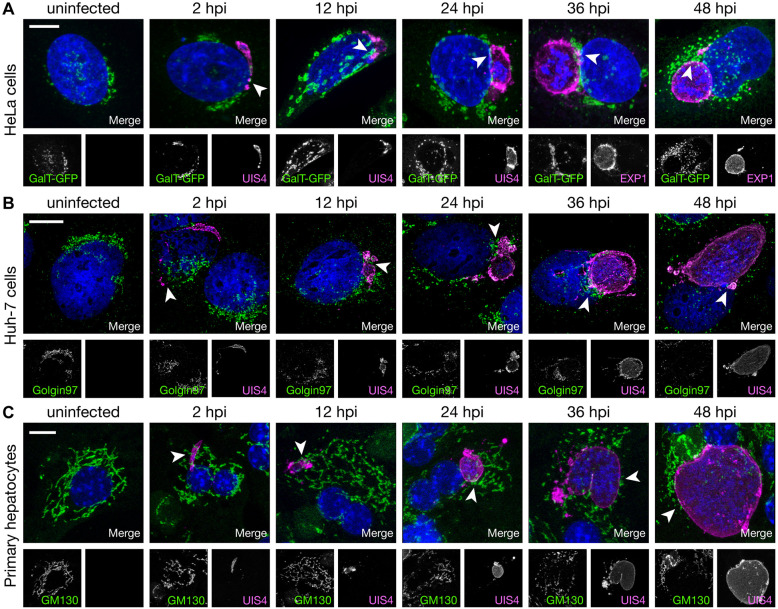
**The PVM of *P. berghei* liver stages associates with *cis-* and *trans-*face of the hcGolgi.** Representative images (3D projections) show the progressive development of *P. berghei* liver stages in different cell lines. Uninfected host cell controls exemplify the morphology of the hcGolgi compartments in the indicated cell lines. In all images, the respective hcGolgi compartment is displayed in green, the PVM of the parasite in magenta and the nuclei stained by DAPI in blue. (A) HeLa cells transiently expressing the *trans*-Golgi marker GalT-GFP. In HeLa cells, the PVM was visualized with antibodies against either UIS4 (2–24 hpi) or EXP1 (>36 hpi). (B) In human hepatoma Huh-7 cells, the *trans*-Golgi was visualized with anti-Golgin-97. (C) In primary hepatocytes the *cis*-Golgi was visualized with anti-GM130. In B and C, the PVM was stained using anti-UIS4. Arrowheads indicate PVM or TVN contact with the hcGolgi. Scale bars: 10 µm.

Using quantitative fluorescence microscopy, we characterized the relationship of *P. berghei* parasites to the hcGolgi during the pre-erythrocytic development. Huh-7 cells transiently transfected with GFP-tagged β-1,4-galactosyltransferase (symbol B4GALT1; GalT-GFP) a marker for the *trans*-Golgi network (TGN), were infected with *P. berghei* sporozoites expressing cytosolic mCherry (PbmCherry). Time-lapse microscopy was performed on an invaded sporozoite settling adjacent to the hcGolgi within the first hour of imaging (Movie 1). For an extended characterization of the parasite interaction with the *cis*- and *trans*-face of the hcGolgi, we infected primary mouse hepatocytes, human hepatoma cells (Huh-7) and human cervical HeLa cells (all well-known to promote the complete pre-erythrocytic development of *P. berghei*) and fixed them at representative time points for antibody staining (Fig. 1). The *cis-*Golgi network (CGN) was visualized by an antibody recognizing the endogenous *cis*-Golgi matrix protein GM130 (also known as GOLGA2). Imaging of the TGN was performed on HeLa cells transiently expressing the *trans*-Golgi enzyme GalT–GFP (Fig. 1A) as well as Huh-7 cells stained for the endogenous Golgin97, a peripheral membrane protein on the cytoplasmic face of the TGN (Fig. 1B). The parasitophorous vacuolar membrane (PVM), in which the parasite develops, and its membranous expansion the tubovesicular network (TVN) was visualized by staining against either the early PVM proteins upregulated in sporozoites 4 (UIS4) or exported protein 1 (EXP1). 3D confocal laser scanning microscopy imaging (3D-CLSM) revealed that there already was an interaction of the proliferating parasite with the different hcGolgi subcompartments 2 h after invasion. The contact to the hcGolgi via different PVM subcompartments is maintained at all time points, independent of the cell line in which the parasite develops, and irrespective of the PVM-resident protein (Fig. 1).

**Fig. 2.**
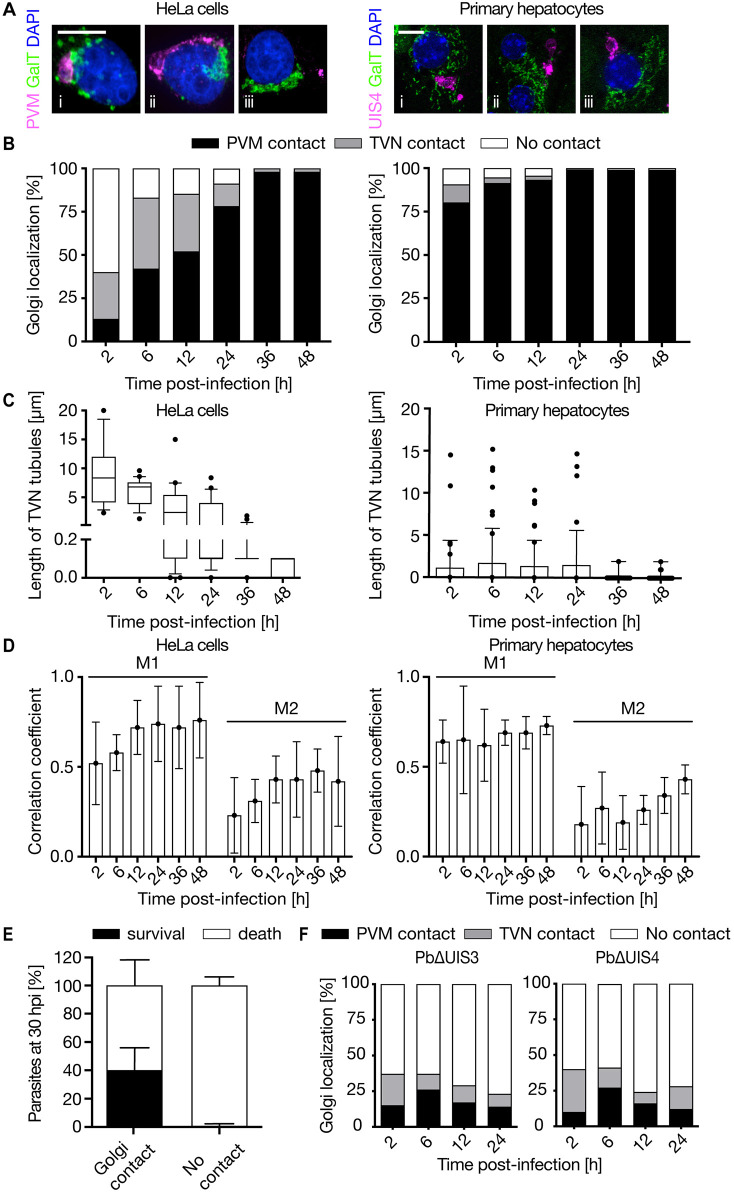
**The PVM and TVN interacts with the hcGolgi over the infection time.** Quantification of the hcGolgi localization relative to the parasite over 48 h of liver-stage development. The interaction is observed in HeLa cells (left panel) and primary hepatocytes (right panel). (A) Representative images (3D projections) of the hcGolgi–parasite interaction at 12 hpi. The hcGolgi (green) was visualized in HeLa cells by transient transfection with GalT–GFP and in the primary hepatocytes by using anti-GM130. The PVM (magenta) was stained with anti-UIS4 and nuclei (blue) with DAPI. (B) Graph indicates the percentage of parasites displaying each type of localization relative to the hcGolgi at different time points of the infection: contact with the PVM [i.e. hcGolgi in close proximity to the parasite body (black)]; contact via the TVN [i.e. contact exclusively via the TVN tubular protrusion (gray)]; or no contact [i.e. neither PVM nor TVN in contact with the hcGolgi (white)]. (C) The length of the TVN extension reaching towards the hcGolgi was quantified at selected times post-infection. (D) The degree of co-localization between PVM and hcGolgi over the infection time was measured by the Mander's overlap coefficient (MOC). M1: portion of PVM overlapping with hcGolgi and M2: portion of hcGolgi overlapping with PVM. Error bars are mean±s.d. (E) Quantification of parasite death-like phenotypes in PbmCherry (30 hpi) associated with or lacking hcGolgi contact. Parasite death defined based on morphological features described by Eickel et al. (2013). (F) Quantification of Golgi-association in PVM-deficient knockout parasites PbΔUIS3 (left graph) and PbΔUIS4 (right graph). Graph indicates the percentage of parasites displaying hcGolgi associations during the first 24 h of infection. Quantitative results are from three independent experiments. Scale bars: 10 µm.

Live-cell recording of a UIS4–mCherry-expressing trophozoite [7 hours post infection (hpi)] in Huh-7 cells transiently expressing GalT–GFP demonstrated a highly dynamic interplay between the parasite and hcGolgi. The *trans*-Golgi elements associated with both the TVN and PVM likewise displayed a similar mobility (Movie 2). To determine whether *Plasmodium*–hcGolgi interaction shows a predominant association via the PVM or TVN, hcGolgi proximity to the PVM was quantified from 2 hpi onwards, up to 48 hpi (Fig. 2). Classification of the contact site was hierarchical, parasites in contact with the hcGolgi via the PVM or multiple contact points with PVM and TVN were classified as ‘PVM contact’. Only parasites showing exclusive contact at the TVN or no contact with the hcGolgi at all were considered as ‘TVN contact’ or ‘no contact’, respectively (Fig. S2A). In HeLa cells, 45% of the invaded sporozoites were already in contact with the hcGolgi at 2 hpi. While a number of parasites in proximity to the hcGolgi showed association via the PVM (12%), the majority of contact events occurred via TVN protrusions (33%), bridging towards the distantly located hcGolgi (Fig. 2Aii). Moreover, hcGolgi proximity to or contact with the parasite rapidly increased over the time of infection. At 6 hpi, 78% of parasites were in contact with the hcGolgi. In the course of the early development of the schizont most parasites had established contact with the hcGolgi. The relationship of the PVM with the hcGolgi was observed during the entire 48 h of liver stage development (Fig. 2B, left panel). In primary hepatocytes, we observed an analogous behavior; however, the association of parasite with the hcGolgi proceeded faster than in HeLa cells. About 90% of the parasites already had contact with either the TVN or PVM at 2 hpi. Fewer parasites showed an exclusive contact through the TVN, which might be due to the high abundance of hcGolgi in primary hepatocytes. Independently of the tested cell line, the hcGolgi is recruited towards the parasite during the first 24 h of the infection. A shift from TVN-association of the hcGolgi towards a PVM localization as infection progresses is observed in primary hepatocytes as well as HeLa cells.

Quantification of the length of the TVN projections associating with the hcGolgi in HeLa showed a progressive shortening of the TVN extensions over time (Fig. 2C, left panel). While TVN extensions of the PVM surrounding invaded sporozoites (2 hpi) displayed a median length of 8 µm and could reach up to 20 µm, from 12 hpi onwards this distribution became significantly reduced, resulting in an absolute minimum of 0.1 µm in mature schizonts (≥38 hpi) (Fig. 2C, left panel). While the hcGolgi contacting with TVN extensions is rather characteristic for the early time points post-infection (2–24 hpi), the PVM–hcGolgi contact becomes predominant at later stages (36–48 hpi). The TVN extensions that interact with the hcGolgi in primary hepatocytes can reach up to 15 µm; a similar maximum length to in HeLa cells. During the first 24 h the median length of TVN protrusion in infected primary hepatocytes was significantly shorter (1–2 µm) than in HeLa cells. In older parasite stages (≥38 hpi) the median length of TVN (0.2 µm) found associated with the hcGolgi became significantly shorter. However, in infected primary hepatocytes the shorting is not as pronounced and continuous as in HeLa cells (Fig. 2C).

To determine whether the surface area of hcGolgi covering the PVM also increases over time, the Pearson's correlation coefficient (PCC) and Mander's overlap coefficient (MOC) was calculated to determine the degree of colocalization between GalT–GFP and the parasite PVM. While PCC was measured in a region of interest (ROI) around the parasite (Fig. S2B), MOC was determined on the entire host cell (Fig. 2D). Through both methods, we observed a progressive increase in the degree of colocalization over time, indicating that more of the hcGolgi aligned to the surface of the PVM. The amount of hcGolgi covering the PVM surface increased independently of the parasite size gain associated with its growth. A considerable portion of hcGolgi, however, was scattered through the host cell cytoplasm. That is particularly true in primary hepatocytes, where the *cis*-Golgi covers a large portion of the host cell cytoplasm (Fig. 2D).

Equally importantly, we observed that parasites without Golgi contact often displayed characteristic features of parasite death. The classification of parasite death is based on morphological abnormalities observed in parasites expressing a fluorescent protein in the parasite cytoplasm: (1) loss of the parasite membrane integrity resulting in leakage of fluorescent protein into the host cell cytoplasm, (2) vacuole formations in parasite cytoplasm and (3) fragmentation of the parasite (Eickel et al., 2013). We visually classified PbmCherry liver schizonts (30 hpi) depending on the association with the hcGolgi. Almost all parasites (99%) that lacked contact with hcGolgi presented morphological criteria of parasite death. When in contact with the hcGolgi, only 40–60% of the parasites displayed a death phenotype (Fig. 2E). As previously published work has shown, in general only 50% of the liver stage parasites survive during the first 30 h of their development (Prado et al., 2015).

Mutant parasites lacking the PVM proteins UIS3 (PbΔUIS3) (Mueller et al., 2005b) or UIS4 (PbΔUIS4) (Mueller et al., 2005a) are surrounded by a PVM with impaired function. To visualize the PVM, PbΔUIS3 and PbΔUIS4 mutants were stained with the anti-EXP1 antibody (Fig. S2C). Because both knockout (KO) lines fail to develop into mature liver stages, parasite numbers beyond the 24 hpi were too few to draw significant conclusions on the interaction. In the first 24 h of infection, WT parasites display the most pronounced increase in hcGolgi contacts with the PVM as described above (Fig. 2A,B). Both PbΔUIS3 and PbΔUIS4 showed significantly diminished hcGolgi contact via TVN protrusions and PVM (Fig. 2F). Altogether, a compromised PVM function seems to have a direct impact on the PVM–hcGolgi interplay.

### Fragmentation of the hcGolgi leads to the formation of PVM-associated mini-stacks

Having determined the preferential localization of the parasite to the vicinity of the hcGolgi, we went on to determine changes in its morphology throughout *P. berghei* infection. Long-term time-lapse imaging of a UIS4–mCherry schizont (20 hpi) in GalT–GFP-expressing Huh-7 cells showed progressive fragmentation of the hcGolgi. We observed a loss of the perinuclear position of the TGN and redistribution of the smaller hcGolgi fragments in the host cell cytoplasm and an accumulation of the fragments at the TVN and PVM (Movie 3). An analogous movie was recorded with a 30 hpi PbmCherry schizont positioned adjacent to the GalT–GFP-positive stack of cisternae. Over time we recognized a repositioning of hcGolgi fragments around the parasite (Movie 4).

**Fig. 3.**
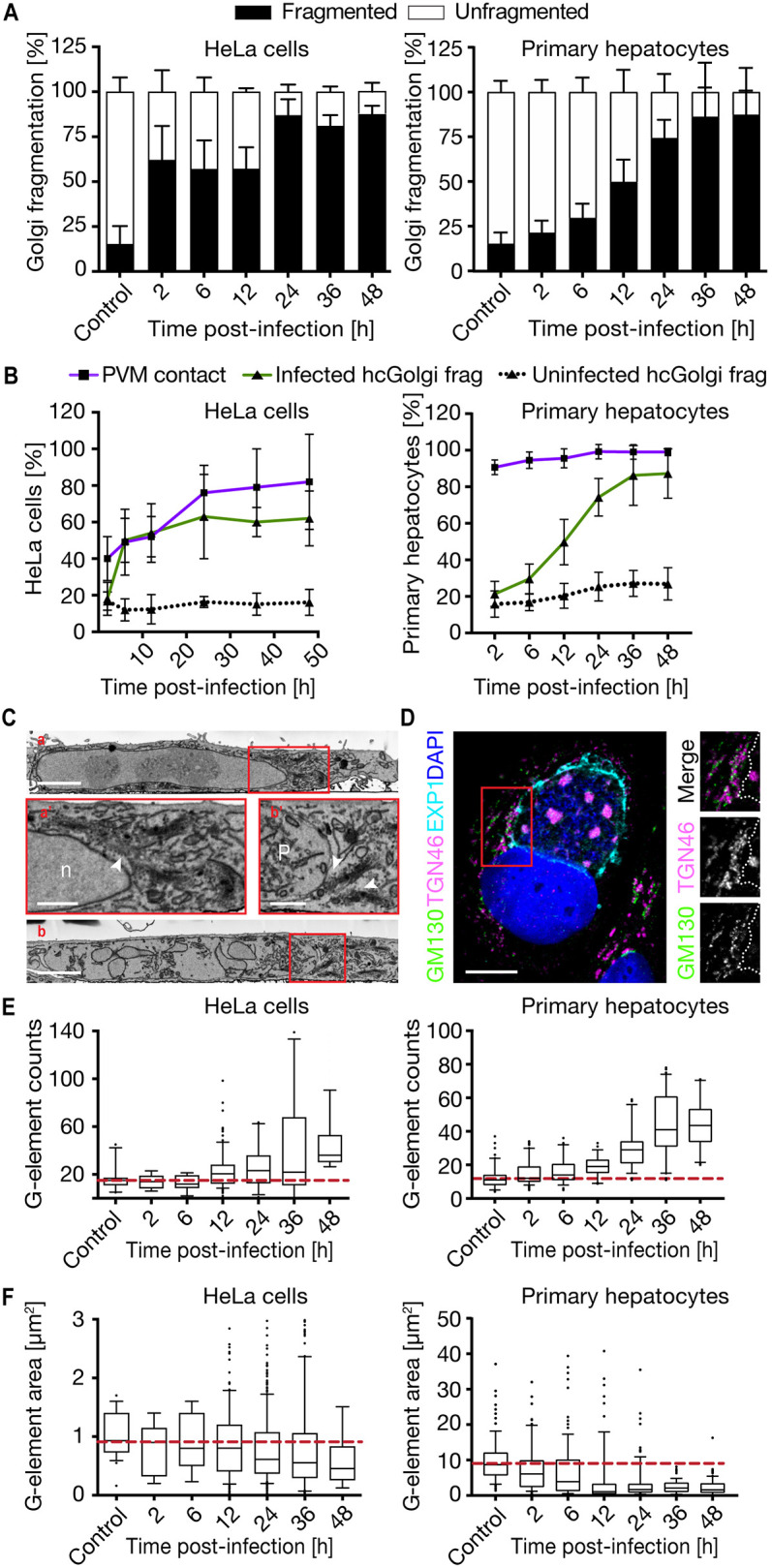
***P. berghei* liver infection promotes hcGolgi fragmentation.** Parasite-induced hcGolgi fragmentation was analyzed in infected HeLa cells transiently expressing GalT–GFP (left panel) and in infected primary hepatocytes by means of anti-GM130 (right panel). (A) Quantification of the parasite-induced fragmentation of the hcGolgi. Percentage of infected cells that display hcGolgi fragmentation, as measured by stack distribution within the cells, and number of Golgi stacks. Uninfected cells were quantified as controls. (B) Comparing the progression of PVM–hcGogli interaction with the hcGolgi fragmentation over the time of infection. In uninfected cells the percentage of cells having a fragmented Golgi remained stable. (C) Serial block face scanning electron microscopy on uninfected HeLa cells (a) and on 48 h-infected cells (b). Red squares indicate the magnified area of hcGolgi apparatus in HeLa cells. (a′) A large hcGolgi at a perinuclear position of the uninfected cell. (b′) Individual hcGolgi complexes with compact stacks of cisternae, located adjacent to the parasite. P, parasite; n, host cell nucleus; white arrowheads indicate the hcGolgi. Scale bars, 3 µm (a and b); 1 µm (a′ and b′). (D) Single-plane confocal image of a schizont (36 hpi) showing individual hcGolgi stacks with a *cis*- (GM130, magenta) to *trans*- (TGN46, green) polarity associated with the PVM (EXP1, turquoise). Red square indicates the magnified area of hcGolgi-PVM association. Dotted line represents the boarder of the PVM. Scale bar: 10 µm. (E,F) Analysis of the degree of Golgi fragmentation during the course of infection. Quantification of Golgi elements (G-elements) numbers (E) and area (F) as infection progresses. All data represent three independent experiments with *n*≥200 infected cells per time point, and are shown as mean±s.d. or box plots as described in the Materials and Methods.

Using 3D-confocal laser scanning microscopy (CLSM) imaging of *P. berghei* fixed at different time points of liver stage development, we confirmed a gradual increased hcGolgi fragmentation over time. This was defined by a wider spread and a higher number of Golgi fragments than in uninfected cells (Figs 1 and 3; Fig. S3). In uninfected host cells, the hcGolgi complex is positioned adjacent to the host cell nucleus (Fig. 1, first panel) and disassembles only when undergoing apoptosis or at the onset of mitosis to ensure correct partitioning into the daughter cells (Petrosyan, 2019). In infected HeLa cells, quantification of hcGolgi fragmentation showed that at early times post-infection (2–12 hpi) more than 50% of infected cells exhibited a fragmented hcGolgi. This proportion increased to over 75% from 24 hpi and was maintained up to 48 hpi (Fig. 3A, left panel). In primary hepatocytes fragmentation of the hcGolgi, evaluated by staining with GM130, was likewise detected, but progressed much more gradually during infection. At 6 hpi the portion of infected cells with a fragmented Golgi was not significantly higher than in the uninfected control, whereas ∼50% of infected primary hepatocytes displayed a fragmented CGN at 12 hpi (Fig. 3A, right panel). The proportion of infected cells with hcGolgi fragmentation correlated directly with time and proximity of the hcGolgi to the TVN and PVM (Fig. 3B). In uninfected host cells, the proportion of cells with fragmented hcGolgi remained constant at 12±10% (mean±s.d.) at all times regardless of the cell type. However, the observed cell-dependent differences in the dynamics of the hcGolgi fragmentation are likely related to the higher abundance of the hcGolgi in primary hepatocytes.

To determine the ultrastructure of the hcGolgi fragments, we performed serial block face scanning electron microscopy (SBF-SEM) on infected HeLa cells (48 hpi) (Fig. 3C). Uninfected host cells possessed a very pronounced hcGolgi occupying a perinuclear position in close proximity to host cell ER. During *Plasmodium* infection, however, the hcGolgi became positioned adjacent to the PVM. Although the hcGolgi appeared smaller in size, the parasite seemed to preserve the typical hcGolgi architecture, consisting of a noticeable stack of cisternae (Fig. 3C). Immunofluorescence analysis of the parasite-associated hcGolgi fragments in Huh-7 confirmed distinct subcellular localization by co-staining for *cis*- (GM130) and *trans*-Golgi (TGN46, also known as TGOLN2) proteins (Fig. 3D). The parasite-dependent fragmentation of the hcGolgi seems to maintain the structural integrity and results in the formation of miniature organelles.

To analyze the degree of fragmentation in HeLa cells and primary hepatocytes over the infection period, we quantified total number and sizes (measured as areas µm^2^) of G-elements in 3D-image reconstructions of uninfected and infected host cells (Fig. 3E,F; Fig. S3). These G-elements are defined as GalT–GFP- or GM130-positive compartments and include different sized vesicles, individual or stacks of cisternae, or even the entire hcGolgi complex (G-element with a median of 1 µm^2^). During the first 12 h of infection, up to 50% of the infected host cells retained the perinuclear localization and ribbon-like morphology of the hcGolgi. This was the case despite the proximity of hcGolgi to the parasite. From 12 hpi, the number of G-elements compared to uninfected cells were about twofold higher and continued to increase. By 48 hpi, infected cells showed three times more G-elements than the uninfected controls (Fig. 3E, left panel). Conversely, while the area of each G-element had a median of 0.9 µm^2^ in uninfected controls, sizes of elements gradually decreased, becoming half the size in host cells infected with parasites at 48 hpi (Fig. 3F, left panel). Progression of hcGolgi fragmentation advanced analogously in the infected primary hepatocytes (Fig. 3E,F, right panels). With a median G-element size of 9 µm^2^ in the uninfected primary hepatocytes the median G-elements are ten times larger than in HeLa cells. During the first 12 h of infection of the primary hepatocytes, the G-element sizes decrease down to 10% of the original. Fragmentation of the hcGolgi in primary hepatocytes is even more pronounced than in HeLa cells and therefore cell-type independent.

Altogether, host cells infected with *P. berghei* display a re-organization and re-distribution of the hcGolgi. The same phenomenon of parasite-induced hcGolgi fragmentation was observed in an independent study in HepG2-CD81 infected with *P. yoelii* (Vijayan et al., 2020 preprint), suggesting a common mechanism during liver-stage development for rodent malaria models.

### The host cell microtubule network supports hcGolgi-parasite interaction and parasite liver stage development

Microtubule (MT) networks play an important role in centralization of the Golgi at a perinuclear location, in maintaining a *cis-trans* polarity and Golgi ribbon assembly, and in ensuring directional vesicle transport (de Forges et al., 2012; Wu and Akhmanova, 2017). To determine the contribution of host cell MT to the hcGolgi–PVM interactions, we altered MT dynamics of infected cells. First, we explored the effect of two easily reversible MT inhibitors, namely nocodazole and Taxol. The minimum concentration of nocodazole or Taxol sufficient to elicit a disruptive effect on the hcGolgi apparatus was determined in uninfected cells by staining against α-tubulin and GM130 (Fig. S4A).

**Fig. 4.**
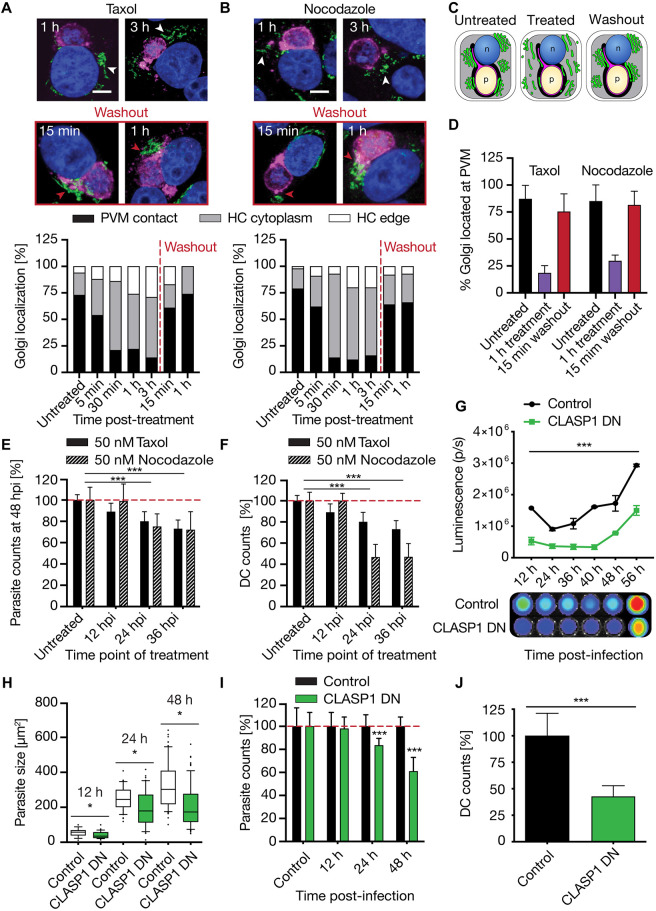
**Interference with host cell MTs affects parasite-hcGolgi interaction and pre-erythrocytic development of *P. berghei***. (A,B) Effect of MT-acting drugs on the interconnection of the hcGolgi with the PVM. HeLa cells transiently expressing GalT-GFP (green) were infected with WT parasites. Schizonts (24 hpi) were treated with 50 nM of Taxol (A) or nocodazole (B) as indicated. To allow MT lattice to recover, drugs were washed out from 3 h-treated infected cells, and hcGolgi positioning determined 15 min and 1 h after wash. The PVM (magenta) was stained using anti-UIS4 or anti-EXP1 and nuclei (blue) with DNA dye, DAPI. White arrowheads point to hcGolgi distributed in the host cell cytoplasm. Red arrowheads highlight the PVM-hcGolgi contact. Scale bars: 10 µm. Lower panels show quantification of hcGolgi position following treatment with 50 nM Taxol (A) or 50 nM nocodazole and after washout (B). Cells were classified according to three categories: (1) PVM contact, (2) host cytoplasm, and (3) cell edges, as illustrated in the schematic (C). (D) Percentage of hcGolgi that remained associated with the PVM in schizonts (24 hpi) following treatment with 50 nM Taxol or 50 nM nocodazole and after recovery of the MT cytoskeleton. (E,F) Impact of MT-dependent cell functions on the development of liver stage parasite. Infected cells were treated once for 1 h with 50 nM Taxol or 50 nM nocodazole at different time points. (E) Percentage of total number of schizonts (48 hpi) relative to untreated cells. (F) Percentage of detached cells (DC) shown relative to untreated cells at 65 hpi. The total number of DCs was normalized to the total number of parasites at 48 hpi. (G-J) Impact of non-centrosomal hcMicrotubules on *P. berghei* liver-stage development. Transient overexpression of the microtubule binding domain of CLASP1, resulting in loss of Golgi ribbon morphology and impaired intra- and post-Golgi cargo transport. (G) Quantification of bioluminescence signals throughout the pre-erythrocytic development of PbNLuc parasites in CLASP1DN and GFP control cells. (H–J) Image-based quantification of the *P. berghei* liver stage development in CLASP1DN or GFP expressing cells, based on parasite size (H) and the relative number of parasites compared to the GFP control (I). (J) Percent of DCs formed (65 hpi) relative to GFP control cells. The total number of DCs was normalized to the total number of parasites at 48 hpi. All data correspond to triplicate experiments and triplicate repeats per experiment, with *n*=200 per cell type, and are shown as mean±s.d. or box plots as described in the Materials and Methods. *P≤0.05, ***P≤0.001 (paired *t*-test or one-way ANOVA as described in the Materials and Methods).

To determine whether host cell MTs alter the localization and distribution of the hcGolgi around the PVM, we treated HeLa cells infected with *Pb*mCherry with 50 nM of the respective drug for up to 3 h. We focused on 24 h parasites when host organelle rearrangement becomes noticeably pronounced and parasite size does not significantly constrain the host cytoplasm space. The degree of hcGolgi dispersion was evaluated based on GalT–GFP fluorescence in relation to the PVM, and infected cells were subsequently classified into the categories: (1) PVM-contact, (2) host cell cytoplasm and (3) host cell edge (Fig. 4A–C). In comparison to untreated control parasites, drug-induced disassembly of the hcGolgi stack can be observed within the first 5 min after addition of either drug. After 30 min of drug incubation only 20% of the infected cells retained a prominent association of the hcGolgi with the PVM or TVN. Longer incubation times caused further scattering of the G-elements throughout the host cytoplasm and a slight increase of the portion of infected cells with G-elements accumulating at the cell edge (Fig. 4A,B). Quantification of the localization of G-elements revealed that a substantial population of G-elements did not dissociate from the PVM in the inhibitor-treated cells. In addition to a MT-dependent parasite–hcGolgi association, the parasite seems to form a MT-independent connection with individual G-elements (Fig. 4D). Upon washout of the drugs, the G-elements reassembled into larger hcGolgi structures within 15 min. In the majority of the infected host cells, this reassembly occurred directly at the vicinity of the PVM (Fig. 4A–D).

To evaluate the importance of the host cell MT cytoskeleton and its associated host cell functions on parasite proliferation, we went on to characterize parasite fitness after interfering with the MT network. Because treatment with nocodazole and Taxol is reversible and allows us to determine the developmental stage that is most vulnerable to MT manipulations, we treated infected cells once for 1 h at 12 hpi, 24 hpi or 36 hpi with either inhibitor. After careful washout of the drug, the parasites were either allowed to develop into 48 h schizonts (total parasite count) (Fig. 4E) or to complete liver stage development [detached cell (DC) formation] (Fig. 4F). While the 1 h inhibitor treatment of young liver stage parasites (12 hpi) did not compromise parasite development, older 24 h and 36 h schizonts were sensitive, particularly to nocodazole (Fig. 4E,F). The divergence on the parasite growth might be due to the different mode of mechanism and kinetics of the MT drugs. Moreover, with this experimental setup we could not exclude that both drugs might also negatively affect the parasite MTs.

To eliminate this potential confounder, we transiently overexpressed the microtubule-binding domain of CLIP-associating protein 1 (CLASP1; domain called CLASP1DN) in HeLa cells, resulting in a dominant-negative phenotype (Maiato et al., 2003a,b). CLASP1 is a microtubule plus-end tracking protein that promotes the stabilization of non-centrosome-derived microtubules. It is essential for the control of Golgi-derived microtubules keeping ribbon morphology, and the intra- and post-Golgi cargo transport functioning (Miller et al., 2009). Transient transfection of host cells with EGFP–CLASP1DN resulted in a dispersal of the hcGolgi in transfected cells (Fig. S4B–D). Subsequent infection with a transgenic PbNLuc *P. berghei* line constitutively expressing NanoLuc luciferase (De Niz et al., 2016) showed a significant decrease of luminescence during the proliferative phase of the parasite (12 hpi onwards) compared to what was seen with control cells (Fig. 4G). Our imaging-based quantitative analysis revealed that the phenotype was due to a reduction in parasite sizes as well as overall parasite numbers (Fig. 4H–J). Furthermore, the loss of CLASP1-mediated Golgi organization and function prevents efficient parasite development and survival, as assessed by DC formation (Fig. 4J). The importance of non-centrosome-derived MT for the parasite development was recently confirmed in *P. yoelii* liver-stages, where they are suggested to be involved in the Golgi-associated vesicle transport to the PVM (Vijayan et al., 2020 preprint).

### Identification of Golgi-associated small GTPases with negative impact on the parasite fitness

**Fig. 5.**
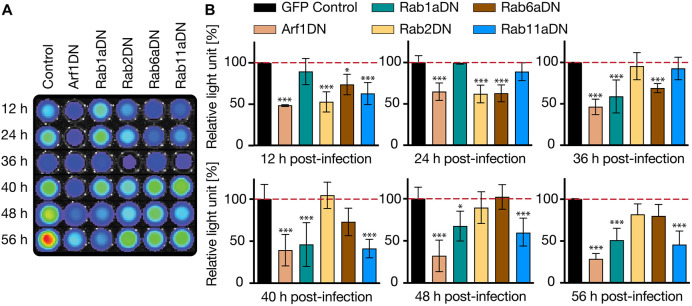
**Bioluminescence-based targeted screening for regulators of Golgi-associated vesicular transport that hamper fitness of *P. berghei* liver stages**. HeLa cells transiently overexpressing either GFP (control), or one of the dominant-negative GTPases (Arf1DN, Rab1aDN, Rab2DN, Rab6aDN and Rab11aDN) were infected with PbNLuc. (A) Cells were lysed at various times post-infection (12 h, 24 h, 36 h, 40 h, 48 h and 56 h) and bioluminescence of each set of cells measured using an IVIS Lumina II system. (B) Quantitative representation of luminescence measurements at every given time during infection. Parasite luminescence at each time point is represented relative to luminescence in GFP control, represented as 100% (dashed line). All data are shown as the mean±s.d. from three independent experiments with duplicate samples per experiment. *P≤0.05, ***P≤0.01 (paired *t*-test).

Since a direct interaction of the hcGolgi with the PVM appears to support progression and survival of the exo-erythrocytic forms (EEFs), we further dissected the importance of the cellular function of the hcGolgi. To subvert hcGolgi maintenance and organization, we interfered with Golgi-associated vesicular traffic by expressing distinct sets of dominant-negative GTPases. The effect on parasite development was quantified first by bioluminescence imaging. We selected five candidates to explore the effects of overexpression of dominant-negative (DN) mutants on *Plasmodium* development. These candidates were the ADP-ribosylation factor (Arf1; a target of brefeldin A), which recruits the COPI complex and regulates ER–Golgi and intra-Golgi vesicle transport (Beck et al., 2009; D'Souza-Schorey and Chavrier, 2006; Gillingham and Munro, 2007; Kawasaki et al., 2005), and four members of the small Rab GTPase family Rab1a, Rab2 (herein referring to Rab2a), Rab6a, and Rab11a, which orchestrate the vesicular traffic at three main locations of Golgi complex. The small GTP-binding proteins Arf1, Rab1a and Rab2 regulate the vesicle transport between ER exit sites and the *cis*-Golgi. DN mutants of the two ER–Golgi Rabs (1a and 2), as well as Arf1 mutant, with the single threonine exchanged to asparagine at position 31 (T31N), cause disassembly of the hcGolgi. The effect on hcGolgi architecture also has major implications on its cellular function. At the *medial*-Golgi, Rab6 mediates retrograde intra-organelle traffic between the Golgi cisternae, while Rab11a is associated with the TGN. Importantly, loss of function mutations for Rab6a and Rab11a only mildly interfere with the structural organization of the Golgi (Fig. S1, Table S1) (Goud et al., 2018; Kjos et al., 2018; Pfeffer, 2017; Short et al., 2005). As an initial assessment, we monitored parasite load in HeLa cells ectopically expressing the DN mutants of Arf1, Rab1a, Rab2, Rab6a or Rab11a, by bioluminescence. Normal parasite growth was assessed in HeLa cells transiently expressing a cytoplasmic GFP reporter (control) (Fig. 5). The inactive Arf1 mutant had the most severe effect on the entire parasite development. At 12 hpi parasite luminescence was already reduced by 50% and did not recover during the ongoing infection. When blocking anterograde ER to Golgi vesicle traffic with Rab1aDN, a decrease in luminescence signal was observed during growth phase of schizonts. A similar trend was observed with Rab11a (40% reduction at 40 hpi), which regulates the exocytic (*trans*-Golgi to the plasma membrane) and endocytic recycling pathway (Goud et al., 2018; Welz et al., 2014). Both Rab2DN and Rab6aDN resulted in only a slight developmental delay during the first 24 h. However, at later time points parasites were able to recover, showing no significant impact on the overall parasite fitness (Fig. 5B). Therefore, we did not proceed with further characterizations of the Rab2 and Rab6a mutants.

### Cellular function and structural integrity of the hcGolgi is important for *P. berghei* development

Based on our initial luminescence-based screening, we decided to further dissect the developmental phenotype of the parasites and also analyse the parasite-induced hcGolgi-fragmentation in cells deficient for Arf1-, Rab1a- and Rab11a-dependent vesicle transport. For the image-based quantifications, PbmCherry-infected HeLa cells were fixed at various time points of development. The changes in Golgi morphology and subcellular location induced by the loss of GTPase activity (Arf1, Rab1a or Rab11a) and by the parasite infection were visualized by staining against the GM130 (Fig. S5A). The average transfection efficiency in HeLa cell was ∼75% with no significant difference between the transfected construct (Fig. S5B).

**Fig. 6.**
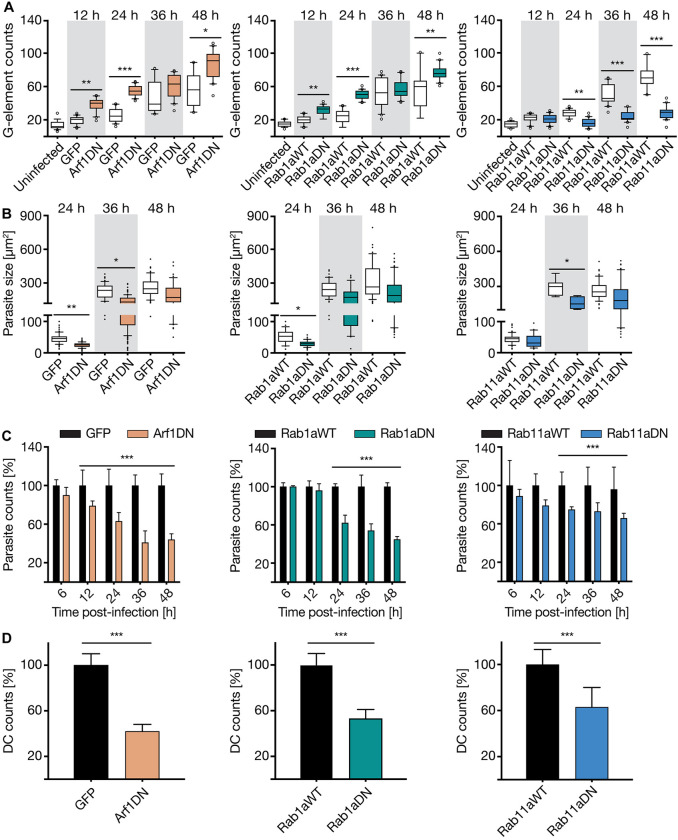
**Image-based characterisation of the parasite development in host cells impaired for Golgi-associated vesicle transport.** In-depth analysis of dominant negative mutants for Arf1, Rab1a and Rab11a. (A) The effect of the respective GTPase mutant on the parasite-induced hcGolgi fragmentation was analyzed based on number of G-elements/cell. Cells overexpressing either cytosolic GFP or the respective WT form of Rab1a or Rab11a served as controls. Expression of GFP or WT constructs had no effect Golgi morphology (uninfected). While Arf1DN (left panel) and Rab1aDN (middle panel) enhance the number of G-element compared to the infected control cells, Rab11aDN (right panel) blocks parasite-induced hcGolgi fragmentation. (B) Parasite growth in Arf1DN (left panel), Rab1aDN (middle panel) and Rab11aDN (right panel) cells was assessed based on parasite sizes at 24, 36 and 48 hpi. (C) Quantification of total parasite numbers in DN cells during the first 48 h of infection. Numbers are shown as a percentage of the control (100%) at each time point. Parasite survival is significantly affected in all three DN mutants. (D) Quantifications of detached cell (DC) formed at 65 hpi relative to WT or GFP control cells, respectively. The total number of DCs was normalized to the total number of parasites at 48 hpi. All data correspond to triplicate experiments and triplicate repeats per experiment, with *n*=200 per time point and transgenic cell line, and are shown as mean±s.d. or box plots as described in the Materials and Methods. *P≤0.05, **P≤0.01, ***P≤0.001 (paired *t*-test or one-way ANOVA as described in the Materials and Methods).

The two small GTPases, Arf1 and Rab1a, tightly regulate opposite vesicle routes between the ER and Golgi (Fig. S1). In both DN mutants, the loss of function results in a fast and complete disassembly of the hcGolgi complex and a loss of hcGolgi homeostasis (Fig. S5C), confirming observations previously shown in other contexts (Donaldson and Honda, 2005; Donaldson et al., 2005; Dascher and Balch, 1994). After infection, we compared the number of G-elements between cells expressing either GFP or wild-type Rab1a (Rab1aWT) as controls, and the respective DN mutant (Arf1DN or Rab1aDN) cells. The G-element counts in infected cells were significantly increased in both mutants at almost all time points. While infected control cells displayed the characteristic parasite-mediated hcGolgi fragmentation, we already detected twice as many G-elements in the Arf1DN and 1.5 times more in Rab1aDN deficient cells at 12 hpi. The phenotype was also observed during schizont growth (Fig. 6A, left and middle panel). Enhanced disintegration of the hcGolgi suggests a cumulative effect triggered by the parasite-induced hcGolgi fragmentation and DN-dependent structural loss of the hcGolgi (Fig. S5C). Because both mutants also display the characteristic hcGolgi disassembly in presence of *P. berghei* infection, we anticipate a loss of its cellular function.

In order to dissect the impact that Arf1DN and Rab1aDN have on *Plasmodium* liver stage development, we measured parasite size (Fig. 6B, left and middle panel; Fig. S6C) and counted total number of parasites at indicated time points (Fig. 6C, left and middle panel; Fig. S6D) and DCs (Fig. 6D, left and middle panel; Fig. S6D). For the quantification of parasite size and numbers, only infected HeLa cells that expressed either the mutant or wild-type GTPase were considered, based on the detected GFP signal. Secondary effects based on differences in parasite invasion efficiency determined at 2 hpi were not observed (Fig. S6A). The intercepted vesicle transport in the host cell affected the parasite development in two aspects. On one hand, we observed a delay in parasite growth, which arose early in the 24-h-old schizonts. On the other hand, we detected a gradual decrease in parasite numbers (Fig. 6B,C, left and middle panel). By 48 hpi, only 50% of parasite survived in comparison to the control cells (Fig. 6C, left and middle panel; Fig. S6D). The decreased survival rate together with the reduction in DCs at 65 hpi indicated a reduced success rate for the parasite to complete development. Relative to the infected GFP control cells, only 40% (Arf1DN) to 55% (Rab1aDN) of parasites completed the liver stage (Fig. 6D, left and middle panel). In HeLa cells transiently overexpressing Arf1WT, we also observed a tendency towards slightly smaller parasites sizes and an associated delay in the formation of DCs when compared to mock-transfected cells (Fig S6C–E). The onset of the parasite death, however, started earlier (at 12 hpi) in the Arf1DN cells (Fig. 6C, left panel). Likewise, we observed different dynamics in hcGolgi disintegration. In Arf1DN cells, the fragmentation was more pronounced leading to a higher number of G-elements than in the Rab1aDN background (Fig. 6A, left panel; Fig. S5C).

Rab11a regulates the vesicular transport at the *trans-*face of the Golgi and coordinates post-Golgi traffic and recycling of endosomes to the cell surface (Goud et al., 2018; Welz et al., 2014). Moreover, previous work has shown that Rab11 disruption only has a small effect on hcGolgi architecture (Rejman Lipinski et al., 2009; Welz et al., 2014) (Fig. S5A,C). When we infected cells overexpressing Rab11aDN, confocal images confirmed the manifestation of the hcGolgi at the PVM. In contrast to the other mutants, the number of G-elements per cell in the Rab11aDN remained consistent throughout infection, showing no increase of hcGolgi fragmentation (Fig. 6A, right panel). Loss of Rab11a function prevents the parasite-induced fragmentation of the hcGolgi. Thus, Rab11a activity seems critical for the ability of the parasites to remodel the hcGolgi.

We continued by analyzing the parasite development in context of Rab11aDN. Parasite size measurements revealed a significant growth delay in schizonts at 36 hpi (Fig. 6B, right panel). A similar trend was observed regarding parasite numbers at ≥24 hpi. Although parasite survival rate progressively decreased, the effect was less pronounced than in Arf1DN and Rab1aDN. About 70% parasites developed into late schizonts (48 hpi) and 40% fewer DCs formed at 65 hpi compared to control cells (Fig. 6C,D, right panels). Therefore, loss of Rab11a function predominantly affects parasite proliferation. At the current state, we cannot distinguish whether the impaired exocytic and endocytic pathway of the host cell or the Rab11aDN-mediated parasite deficiency to induced Golgi fragmentation are the main cause of the reduction of parasite fitness in the liver.

## DISCUSSION

Following invasion and the formation of the PV, *P. berghei* preferentially settle at the juxtanuclear region of the hepatocyte and shortly afterwards associate with the host cell ER (Bano et al., 2007; Kaiser et al., 2016). Although *P. berghei* interactions with the host cell ER have been investigated, the dynamics and relevance of hcGolgi–*Plasmodium* interactions remained largely unexplored. In the present work, we characterize the parasite relationship with hcGolgi during pre-erythrocytic stages.

The PVM–hcGolgi interplay was sustained during the pre-erythrocytic development and was accompanied by fragmentation of the hcGolgi into miniaturized complexes. hcGolgi fragmentation is certainly not unique to *Plasmodium.* Several bacteria (Allgood and Neunuebel, 2018; Brumell and Scidmore, 2007), as well as various viruses (Ravindran et al., 2016; Spriggs et al., 2019), and the parasite *Toxoplasma* *gondii* (Romano et al., 2017) induce hcGolgi fragmentation by mechanisms including molecular mimicry and modification of hcGolgi proteins. Golgins and Rab GTPases are common targets. In *P. berghei*, fragmentation becomes pronounced only from 24 h onwards corresponding with the initiation of parasite schizogony. At this point, hcGolgi fragments distribute around the PVM, losing in many cases the perinuclear localization.

Work on *T. gondii* has shown that a preferential localization of the parasite near to the microtubule organizing center (MTOC)–Golgi region, which is the intersection of endocytic and exocytic pathways, may facilitate the interception of vesicular trafficking, ensuring acquisition of lipids, such as cholesterol deriving from lysosomes, or ceramide deriving from the hcGolgi (Bisanz et al., 2006; Coppens et al., 2006; Melo and Souza, 1996). Although our approach does not give insight into metabolites scavenged by *P. berghei*, we show that the parasite–hcGolgi interaction plays a critical role for a productive passage through the liver. Extrinsic interference with host cell MTs or hcGolgi-associated vesicular transport impaired the pre-erythrocytic development of the parasite and reduced the number of DCs. Nutrient scavenging is a well-established role of the PVM. Parasite-encoded PVM proteins have been shown to directly interact with lipid-binding proteins and to sequester host lipids by hijacking endo-lysosomal vesicles (Petersen et al., 2017; Sá e Cunha et al., 2017). An alternative route represents the transport of host cell nutrients through pores and channels in the PVM (Bano et al., 2007).

*T. gondii* localizes not only to the periphery of the hcGolgi, but also near to the host MTOC. In polarized and non-polarized cells, the *T. gondii* PV first associates with the hcGolgi, and only later with the MTOC (Romano et al., 2013). We investigated whether inducing hcGolgi ribbon disassembly and vesiculation by interfering with microtubule polymerization would cause complete or partial dissociation of the hcGolgi from the PVM. The effect of each drug on microtubule conformation was consistent with previous observations (van de Moortele et al., 1993). Both drugs induced hcGolgi disassembly and dispersion across the cytoplasm in a reversible manner. While nocodazole is a MT-depolymerizing drug giving rise to mini-stacks adjacent to the ER exit sites, Taxol acts as MT-stabilizing agent that supports the polymerization of straight MT bundles and redistribution of G-elements in the peripheral cytoplasm enriched for MT. Upon washout of the drugs, the Golgi re-distributed as a fragmented structure around the PVM, rather than re-stacking as a single Golgi ribbon in the nuclear periphery of the host cell. Even after prolonged incubation with the drugs, individual G-elements did not fully detach from the PVM. The drug-resistant G-elements seem to form stable, MT-independent attachment with PVM. Similar contacts were previously described for host cell ER with the PVM (Kaiser et al., 2016), suggesting the presence of membrane contact sites. The nature and cellular function of the Golgi–PVM attachment will be subject of future investigations.

Crucial for establishing the hcGolgi–parasite interaction is the presence of a functional PVM. Mutants missing the PVM-resident proteins UIS3 or UIS4 are surrounded by a functionally compromised PVM (Mueller et al., 2005a,b). Both PVM-deficient parasites are not able to recruit or maintain a stable connection with hcGolgi. Host cell vesicles of the endo-lysosomal and of the autophagy pathway are engaged in a more spatio-temporal restricted relation with the PVM and its dynamic TVN extensions. This carefully controlled association guarantees optimal nutrient acquisition and simultaneously prevents the elimination of parasite (Agop-Nersesian et al., 2018, 2017; Grützke et al., 2014; Niklaus et al., 2019; Petersen et al., 2017; Prado et al., 2015; Real et al., 2018; Thieleke-Matos et al., 2016; Wacker et al., 2017). With the hcGolgi, however, parasites build a persistent relationship. Loss of the hcGolgi contact is accompanied with morphological features of parasite death, suggesting that the interaction plays a role beyond nutrient scavenging.

Strategies to overcome host defenses developed by pathogens, include molecular mimicry (Spanò and Galán, 2018), inhibition of signal transduction cascades involved in pattern recognition receptor (PRR) activation (Espinosa and Alfano, 2004; Mogensen, 2009), inhibition of antigen presentation, or inhibition of vesicular transport through the secretory pathway of the host, with the purpose of abrogating chemokine or cytokine secretion (Burnaevskiy et al., 2013; Dong et al., 2012; Selyunin and Alto, 2011). Cargo in the secretory pathway usually follows a route including the ER, the ER Golgi intermediate compartment (ERGIC) and the Golgi. Arf and Rab family GTPases are essential for cargo packaging and delivery between compartments. Various pathogens particularly target members of the Rab and Arf families to exploit nutrient hijacking. We investigated the role of Arf1 on *Plasmodium* development, as well as representative Rab GTPases with functions at various points of the trafficking pathway (Fig. S1, Table S1).

Arf1 plays a key role in the early secretory pathway, primarily with regards to retrograde transport from the Golgi to the ER and between Golgi cisternae via recruitment of the COPI to budding transport vesicles (Bonifacino and Glick, 2004; Singh et al., 2019). Among various candidates tested, Arf1DN was the most detrimental for *Plasmodium* development, with deleterious effects on parasite sizes and numbers detected since very early on in infection. Despite equally successful sporozoite invasion rates to those in cells expressing the WT GTPase, parasites show poor survival, development, and ultimately relatively unsuccessful completion of the pre-erythrocytic stage. Indeed, the downregulation of vesicular coat subunits (COPB2 and COPG1) in the COPI complex have also shown to impair *Plasmodium* development in the liver (Raphemot et al., 2019). Moreover, the β-COP subunit vesicles also localized to the PVM in late schizonts. Various pathogens have previously been shown to target Arf1, including *Legionella pneumophila* and *E. coli*. *Legionella* activates and recruits Arf1 to establish vesicular trafficking of the hcGolgi to the phagosome, providing the latter with ER-like characteristics which favor bacterial development (Amor et al., 2005; Nagai et al., 2002). Likewise, *E. coli* simultaneously inhibits Arf1 and Rab1, while Arf1-binding-deficient mutants are unable to disrupt Golgi architecture (Selyunin et al., 2014).

The tested Rab proteins included the candidates Rab1a and Rab2, both of which are localized to the ER/*cis*-Golgi region and are involved in ER-Golgi transport; Rab6a, which localizes to the Golgi and is involved in retrograde Golgi traffic, and Rab11a, which localizes to the recycling endosomes and the *trans-*Golgi network (Goud et al., 2018). Among these, the most striking effects on *Plasmodium* development were observed with Rab1a and Rab11aDN, while overexpression of Rab2aDN and Rab6aDN had no overall impact on parasite fitness. The parasite-induced increase of the surface area and contact sites of the hcGolgi with the PVM is beneficial for parasite development, while artificial disassembly of the hcGolgi either by microtubule manipulation or upon expression of Arf1DN or Rab1DN is detrimental for the parasite. We hypothesize that the type of fragmentation induced by the parasite preserves hcGolgi function, at least partially. Conversely, Rab1a or Arf1DN overexpression dismantle the Golgi complex. Regarding Rab11, previous work on *Chlamydia trachomatis* had shown that both Rab6 and Rab11 are important regulators of infection. Their disruption prevented *Chlamydia*-induced Golgi fragmentation, which resulted in the inhibition of transport of nutrients to bacterial inclusions (Rejman Lipinski et al., 2009). In our work, we observed similarly, reduced hcGolgi fragmentation with Rab11aDN. In contrast to Rab6aDN, Rab11aDN caused significant effects on parasite development, suggesting that functions specific to Rab11, beyond hcGolgi fragmentation, are important. Rab11a localizes to recycling endosomes, and is key for anterograde trafficking from the TGN to the plasma membrane. Its inhibition has previously been shown to result in reduced surface delivery of cargo, while cargo trafficking from ER to Golgi and intra-Golgi remained unaffected (Welz et al., 2014). Together with the clathrin adaptor protein GGA1, Rab11a represents an additional host cell factor involved in the *trans*-Golgi to endosome vesicular transport (Raphemot et al., 2019).

With host-targeted therapies becoming a new line of intervention, understanding the nature of the host–parasite interaction is of great importance. In this study, we not only describe the dynamics and structural changes of the hcGolgi upon infection but also shed some light on the importance of the hcGolgi in *Plasmodium* pre-erythrocytic development. We envisage that future work will give further interesting insights into the role the hcGolgi might play in nutrient scavenging and parasite survival.

## MATERIALS AND METHODS

### Ethics statement

Animal studies were carried out under the approval of the Animal Research Ethics Committee of the Canton Bern, Switzerland (Permit Number: BE91/11 and BE81/11, BE86/19); and the University of Bern Animal Care and Use Committee, Switzerland. For all studies (except primary hepatocyte isolation, see below), Balb/c females of 5–8 weeks of age, weighing 20 to 30 g at the time of infection were used. Mice were bred in the central animal facility of the University of Bern or supplied by Harlan Laboratories. Blood feeding of mosquitoes was performed under Ketasol/Dorbene anaesthesia, and all efforts were made to minimize suffering.

### *P. berghei* lines

Various *P. berghei*-ANKA lines were used to infect mice and cultured cells. Transgenic lines used in this study included Pb^Hsp70^mCherry (referred to as PbmCherry), which constitutively expresses mCherry in the parasite cytosol (Burda et al., 2015); Pb ^Hsp70^mCherry ^ef1α^NLuc (referred to as PbNLuc), which constitutively expresses mCherry and the luminescent protein NanoLuc (De Niz et al., 2016); Pb^Lisp2^Exp1-mCherry (Graewe et al., 2011), which expresses mCherry-tagged EXP1 under the liver stage-specific Lisp2 promoter, enabling visualization of the PVM from 36 hpi onwards; and Pb^UIS4^ UIS4-mCherry (Grützke et al., 2014), which expresses the mCherry-tagged PVM protein UIS4 under its endogenous promoter and enables PVM visualization from early times post-infection. We also used the parasite mutants PbΔUIS4 and PbΔUIS3 (Mueller et al., 2005a,b).

### Parasite maintenance in mosquitoes

Balb/c mice were treated with phenylhydrazine 3 days prior to intra-peritoneal infection with the different *P. berghei* lines. After 3 days of infection, parasites were spotted on a coverslip, and exflagellation was determined. The infected mice were then used to feed 100–150 *Anopheles stephensi* female mosquitoes each. Mice were anaesthetized with a combination of Ketasol (125 mg/kg body weight) and Dorbene (12.5 mg/kg body weight) anaesthesia or with a combination of Ketasol (143 mg/kg body weight) and Xylasol (19.3 mg/kg body weight), and euthanized with CO_2_ after completion of the feed. Afterwards, mosquitoes were fed until use with 8% fructose and 0.2% para-aminobenzoic acid (PABA). Sporozoites used for hepatocyte infection were isolated form the salivary glands of mosquitoes between 16 and 26 days post-feed.

### Mammalian cell culture and infections

The human hepatoma Huh-7 cell lines (European Collection of Cell Culture) and the human epithelial HeLa cell lines (kind gift from Robert Menard, Malaria Infection and Immunity Unit, Institute Pasteur Paris, France) were maintained in minimum essential medium (MEM) containing Earle's salts supplemented with 10% heat-inactivated fetal calf serum (FCS), 2 mM L-glutamine, and 100 U penicillin, 100 µg/ml streptomycin (cMEM) (all from PAA laboratories; E15-024, A15-101, P11-010, M11-004). Cells were kept at 37°C in a 5% CO_2_ cell incubator and were split every 4 day by treatment with accutase. For infections (except for high-content imaging, see below), 20,000 cells were seeded into 24-well plates and infected with *P. berghei* sporozoites with an multiplicity of infection (MOI) of 1. Infected cells were maintained in cMEM and 2.5 µg/ml amphotericin B (AT-MEM) (PAA laboratories; P11-001) to avoid fungal contamination. AT-MEM was exchanged every 12 h. Selected time points for assaying infection were 6, 12, 24, 36, 48 and 56 hpi.

### Isolation of primary mouse hepatocytes

Primary hepatocytes from 12–16-week-old Balb/c or C57BL/6 mice were isolated as described previously (Prado et al., 2015). Briefly, immediately after euthanizing the mouse, the liver was sequentially perfused via the portal vein with HEPES buffer for 10 min and 100 U/ml collagen prewarmed to 37°C. Afterwards, the inferior vena cava was incised to drain the blood, circulating cells and the perforate from liver. The inferior vena cava was periodically clamped for 10 s to ensure optimal distribution of the perfusion solutions. From the excised liver, the tissue capsule surrounding the liver was disrupted to release liver cells into wash medium (William medium E containing 2 mM L-glutamine) by gently shaking. The hepatocytes were separated from other liver cells by centrifugation at 50 ***g*** for 2 min and washed two more times in wash medium at same centrifugation settings. Viability of the isolated hepatocytes was assessed using Trypan Blue dye exclusion. 100,000 to 200,000 primary hepatocytes were seeded on cover slips and cultured in William medium E containing 2 mM L-glutamine, 10% FCS and antibiotics (100 U penicillin and 100 µg/ml streptomycin) at 37°C and 5% CO_2_. Before continuing with *P. berghei* infection on the next day, the growth medium was changed.

### Plasmids

The plasmid pEGFPN1-GalT (plasmid #11929) deposited by Jennifer Lippincott-Schwartz (Cole et al., 1996), pcDNA HA Arf1 DN T31 N (plasmid #10833) deposited by Thomas Roberts (Furman et al., 2002) and Arf1 T31N-EGFP (Arf1 DN) (plasmid #49580) deposited by Marci Scidmore were obtained from Addgene. The Arf1 WT-EGFP control plasmid was a gift from Isabelle Derré (Dept. of Microbiology, Immunology, and Cancer Biology, University of Virginia, VA, USA). Plasmids pECFP-C1-Rab1a WT, pECFP-C1-Rab1a DN (AS mutation), pECFP-C1-Rab2 WT, pECFP-C1-Rab2 DN, pECFP-C1-Rab6a WT, pECFP-C1 Rab6a DN, pECFP-C1-Rab11a WT and pECFP-C1-Rab11a DN were provided by W. D. H. Plasmid EGFP-CLASP1 DN overexpressing the microtubule-binding domain was a kind gift from Helder Maiato (Universidade do Porto, Portugal).

### Transient transfection of mammalian cells

For each transfection 5×10^5^ cells were re-suspended in 100 µl filter-sterilized transfection buffer containing 120 mM Na-phosphatase, 5 mM KCl, 20 mM MgCl_2_, 5 mM NaCl pH 7.2 and mixed with 2.5 µg of the respective plasmid. Cells were electroporated using the T-028 program of a Nucleofactor™ 2b transfection device (Lonza). For the immunofluorescence analysis ∼10% of the transfected cells were seeded in each well of a 24-well plate, and glass bottom dishes (MatTek) for live-cell imaging were prepared with a one sixth of the total transfection.

### Treatment with microtubule-targeting drugs nocodazole and Taxol

Following infections with PbmCherry sporozoites, cells were treated with 50 nM nocodazole (M1404, Sigma-Aldrich) or 50 nM Taxol (T7402, Sigma-Aldrich) for 1 h at 24 hpi. For recovery experiments, cells were washed three times with AT-MEM, and fixed after 15 min or 1 h, respectively. For analysis of parasite development, infected cells were treated for 1 h with 50 nM nocodazole or Taxol, respectively, at three different time points, 12 hpi, 24 hpi and 36 hpi. After treatment, cells were washed three times with AT-MEM, and incubated again on AT-MEM until fixation with 4% PFA. Cells were fixed at 48 hpi, after which parasite numbers and sizes in wells were imaged and quantified using the Fiji image analysis software (http://fiji.sc/Fiji). Additionally, at 65 hpi, DCs were quantified. Control cells for each time point were treated with the drug solvent DMSO.

### Immunofluorescence assays

Infected Huh-7, HeLa cells or primary mouse hepatocytes were fixed at the indicated time points either in ice-cold methanol/acetone (1:1) at −20°C for 5 min without further permeabilization step or in 4% paraformaldehyde (PFA) in phosphate-buffered saline [PBS, 137 mM NaCl (Sigma-Aldrich, S9888-1Kg), 2.7 mM KCl (Fluka Chemie AG P9541-1 kg), 10 mM Na_2_HPO_4_ (Sigma-Aldrich, S5136-500 g), 1.8 mM KH2PO4 (Sigma-Aldrich, P5655), pH 7.4] for 10 min at room temperature and subsequently permeabilized with 0.1% Triton X-100 in PBS (Fluka Chemie, T8787-250 ml), for another 10 min. After 1 h blocking with 10% FCS-PBS, cells were stained with the diluted primary antibodies for an additional hour. For fluorescent labeling, cells were subsequently incubated with secondary antibodies for 1 h and nuclei visualized with 1 µg/ml DAPI (Invitrogen, D-1306). All incubations were performed at room temperature. For microscopy, cells were mounted on microscope slides using Dako Fluorescent Mounting Medium (Dako, S3023).

The primary antibodies used were: mouse monoclonal anti-GFP (Roche, 11814460001), rabbit polyclonal anti-GFP (Acris, SP3005P), rabbit polyclonal anti-UIS4 (kindly provided by Photini Sinnis, Johns Hopkins Bloomberg School of Public Health, Baltimore, USA), chicken polyclonal anti-Exp1 (Heussler lab, Bern, Switzerland), mouse monoclonal anti-GM130 (BD-Biosciences, 610822), mouse monoclonal anti-Golgin-97 (Invitrogen, #A21270), rabbit polyclonal TGN46 (abcam #ab16059), mouse monoclonal anti-α tubulin (Sigma, T9026), and rat monoclonal anti-HA (Roche, 11867423001). All primary antibodies were used at a dilution of 1:500 in 10% FCS in PBS. As secondary antibodies, species-specific antibodies conjugated to Alexa Fluor^®^ 488 (Invitrogen Molecular Probes, A-11001; A-11008), Alexa Fluor^®^ 594 (Invitrogen Molecular Probes, A-21209; A-11032) or Cy5 (Dianova, 111-175-144; 703-175-155) were used at a final concentration of 0.2 µg/ml.

### Live cell imaging and time-lapse microscopy

Confocal microscopy was performed on fixed and live cells with a SP8-STED Leica microscope. 3D images were acquired using a 63× oil objective (NA 1.4) and the LAS X software (Leica) used for image acquisition. Optical *z*-sections with 0.3–0.5 µm spacing were acquired using the LAS X software. Time-lapse microscopy on live cells was performed using a Leica DMI6000B microscope equipped with a 63× water objective (NA 1.4), and the Leica Application Suite (LAS) AF software.

### Serial block face scanning electron microscopy

5×10^4^ HeLa cells were seeded in every well of a 96-well plate and infected with PbmCherry parasites, as described above. Infected cells were FACS-sorted at 6 hpi for PbmCherry-infected cells and re-seeded at a density of 10,000 sorted cells per well into a 96-well optical plate (Greiner BioOne). Following this, parasites were fixed in a glutaraldehyde buffer at 48 hpi and processed as per previously published protocols (Deerinck et al., 2010; Kaiser et al., 2016). SBF-SEM images were acquired with a Quanta FEG 250 (FEI Company) equipped with a Gatan 3View2XP ultramicrotome (accelerating voltage=3.5 kV; low vacuum). Images were processed using Fiji.

### Bioluminescence imaging

HeLa cells transiently expressing the indicated plasmids, were infected with PbNLuc (De Niz et al., 2016) sporozoites. At different times post infection, cells were detached from the wells using accutase, and collected and lysed in 20 µl of 1× passive lysis buffer (PLB) (Promega) for downstream analysis. Lysates were transferred to 96-well black plates (Greiner BioOne), and imaged using an IVIS Lumina II system. For consistent and unsaturated measurements, the optimal NanoGlo™ (Promega) assay substrate dilution used for probing liver stage parasites was 1:500. Uninfected cells were used as background controls.

### Analysis of the parasite development by high-content imaging

For analyzing the impact of the Arf1 dominant negative on the parasite growth and survival, we transiently transfected 10^6^ HeLa cells with 1 µg Arf1WT–GFP or Arf1DN–GFP. Mock-transfected HeLa cells served as controls. At 12 h after transfection, multiple wells of a 96-well plate were seeded with 40,000 cells per well. The next day, confluent host cell layers from two wells of each transfection were infected with sporozoites from salivary glands pooled from six mosquitoes. Parasites were left to invade the confluent host cell layer for 2 h. To avoid over-confluency during the next 65 h of parasite development, the cells of each infection were reseeded into 16 individual wells on four µCLEAR 96 well plates (Greiner BioOne; one plate for each time point). At 6 hpi, 24 hpi and 48 hpi the cells were fixed with 4% PFA in PBS and imaged with a 10× air objective on INCell Analyser (GE Healthcare). DCs were transferred into a fresh 96-well plate and imaged. Transfected HeLa cells were identified based on the GFP signal. Parasite number and sizes were measured in the GFP segmented cells using the software INCell Developer Toolbox 1.10.0.

### Image analysis

The shortest distance between the PVM and the hcGolgi was measured using Imaris software on a 3D reconstructed cell volume. Distances are displayed in a box plot, and whiskers show the 10th–90th percentile of the distances measured in 100 cells at each time point. All results appearing as individual dots fall over or under this remaining 10th percentile. No outliers were excluded from calculations or *P-*values.

The number and volume of GalT stacks present in *Plasmodium-*infected and uninfected cells were measured on 3D reconstructed images using Imaris software (Bitplane) and Fiji. Individual foci were identified by intensity, using automatic thresholding. Parasite sizes were quantified using Fiji, and automatic fluorescence thresholding.

### Statistical analyses

Data are displayed in box plots or histograms generated using PRISM 6.0 software. Means, medians, interquartile ranges and s.d. were calculated from three independent experiments performed in triplicate, using STATA 13.0 software. In all experiments a minimum of 100 cells per time point per condition was quantified. The *P-*values were calculated using two-tailed, paired Student's *t-*tests or one-way ANOVA in STATA 13.0. For all graphs, significant values are displayed as follows: *P≤0.05, **P≤0.01, ***P≤0.001.

## Supplementary Material

Supplementary information
