# Supplementary material for: Hijacking of the host cell Golgi by Plasmodium berghei liver stage parasites
Source: J Cell Sci. 2021 May 20;134(10):jcs252213. doi: 10.1242/jcs.252213 (PMC8186485; doi:10.1242/jcs.252213)
Supplement: Supplementary information [file joces-134-252213-s1.pdf]

Supplementary Figures

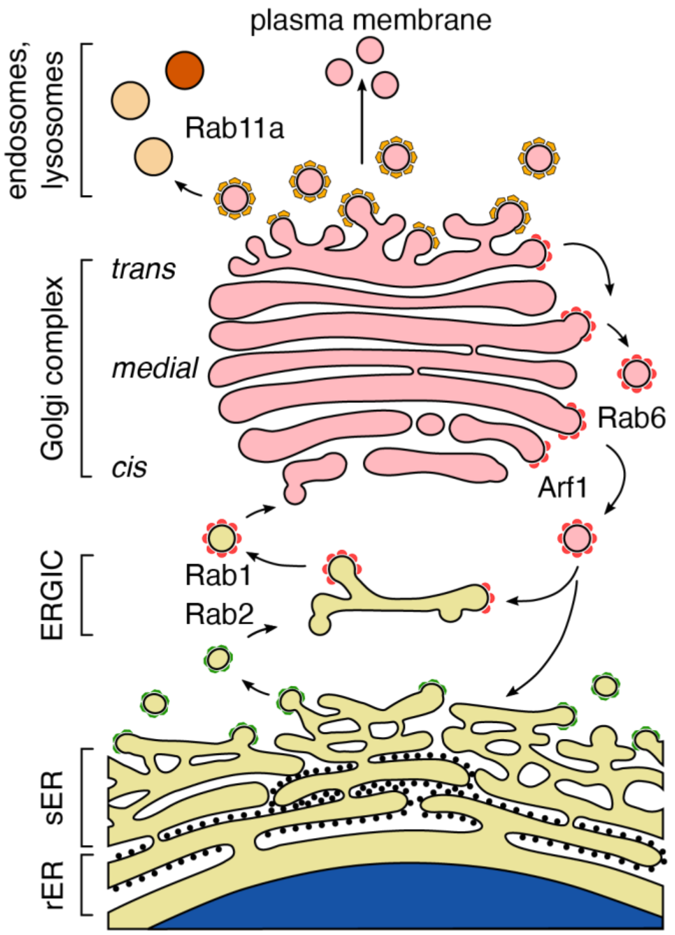

Figure S1. Simplified schematic of the endomembrane system in mammalian cells. Continued vesicle trafficking between the endoplasmic reticulum (ER) and the Golgi apparatus is important for Golgi biogenesis and maintenance. The two major sub-compartments of the ER are the rough ER (rER), composed of interconnected flattened sheets studded with ribosomes, and the entangled tubular network of the smooth ER (sER), where coatomer protein complex 2 (COPII) coated vesicles assemble at the ER exit sites (ERES) (anterograde transport shown on the left side of the schematic). COPII vesicles bridge the 300 to 500 nm space between the ER and the ER Golgi intermediate compartment (ERGIC), which is the first post-ER sorting compartment. Both Rab GTPases 1 and 2 coordinate the anterograde transport from the ER via the ERGIC towards the cis-Golgi. Rab1 regulates the post-ERGIC vesicle transport. There is evidence that COPI coated vesicles sustain the anterograde transport from the ERGIC to the cis-Golgi. The COPI vesicles play a well-established role in the retrograde intra-organelle traffic between the Golgi cisternae as well as for the transport from Golgi and ERGIC back to the ER (right side of the schematic). The COPI components are recruited by the ADP-ribosylated factor 1 (Arf1). At the medial-Golgi the retrograde travel of vesicle is mediated by the small GTPase Rab6. Based on the Golgi maturation model, the cisternae at the cis face of the Golgi mature to cisternae of the trans-Golgi network (TGN). At the trans face, the secretory side of the Golgi, Clathrin-coated vesicle bud off and are either exocytosed at the plasma membrane or targeted to the endo-lysosomal recycling compartment via Rab11.

Journal of Cell Science • Supplementary information

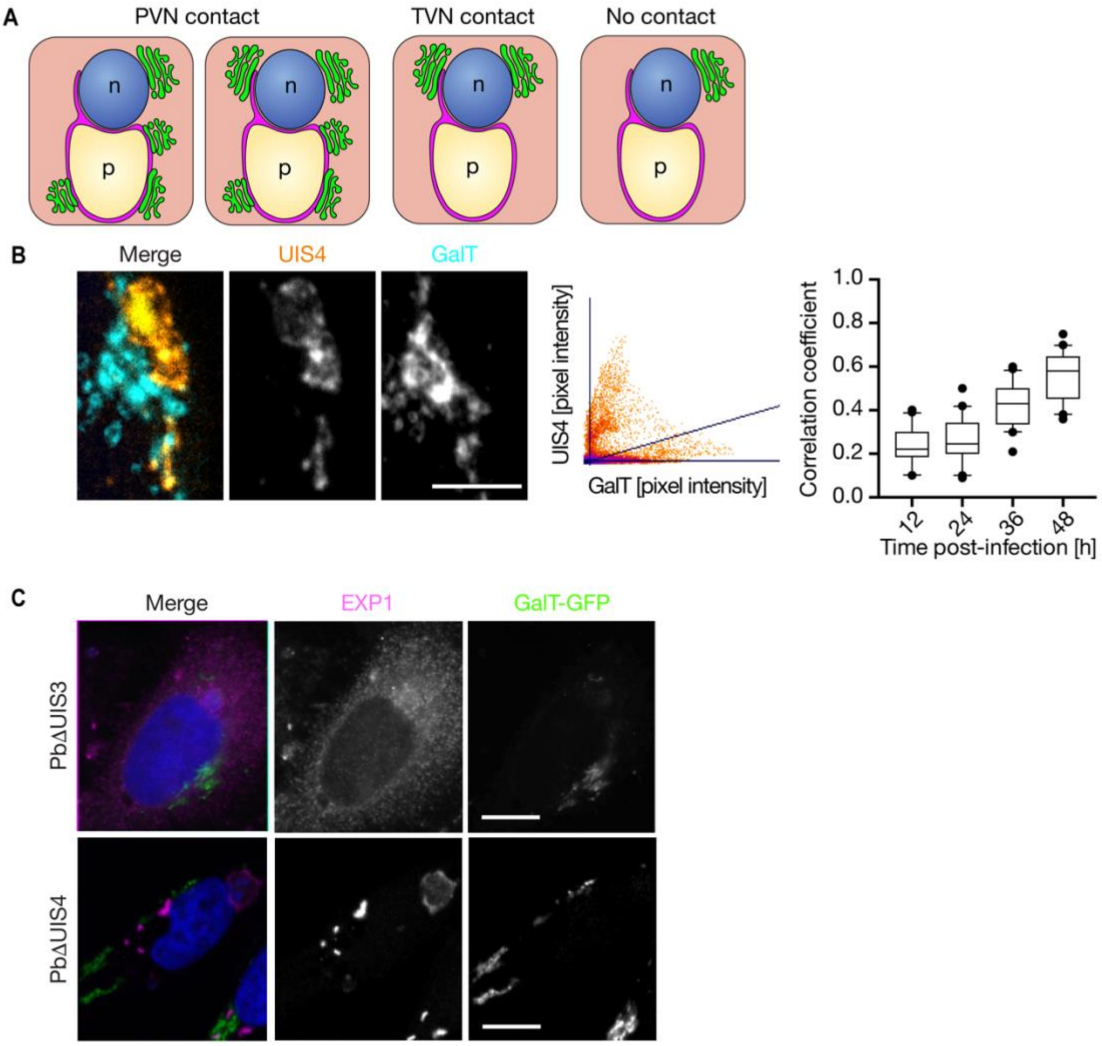

Figure S2. Role of the PVM for the association with the hcGolgi. A) Schematic of the hierarchical classification of the parasite-hcGolgi contact site. PVM contact: exclusive contact of the PVM with the hcGolgi or multiple contact points at the PVM and TVN. TVN contact: exclusive contact of the TVN with the hcGolgi. No contact: neither PVM nor TVN is in contact with the hcGolgi. **B)** Progressive accumulation of hcGolgi at the PVM. Co-localization was measured at the area of contact based on the Pearson's correlation coefficient (PCC), by using the Coloc2 plugin available in Fiji, and confirmed with the co-localization threshold. A PCC value of 0 reflects an uncorrelated distribution of two fluorescence singles, while a value of 1 shows perfect linear relation. Left panel: A representative image of an infected HeLa cell (12 hpi) expressing the *trans*-Golgi protein GalT-GFP (turquoise) that was stained for the PVM marker UIS4 (yellow). Middle panel: PCC analysis of the corresponding image displayed as scatterplot. Right panel: Box plot displays the correlation coefficient of 20 images per time point. **C)** A functional PVM is important for association in with the hcGolgi. Representative images of PVM-deficient knockout parasites PbΔUIS3 (upper panel) and PbΔUIS4 (lower panel) at 24 hpi. The PVM was stained with antibody against EXP1 (magenta). The hcGolgi of HeLa cells was visualized by transfection with GalT-GFP (green). Scale bars, 10 µm.

Journal of Cell Science • Supplementary information

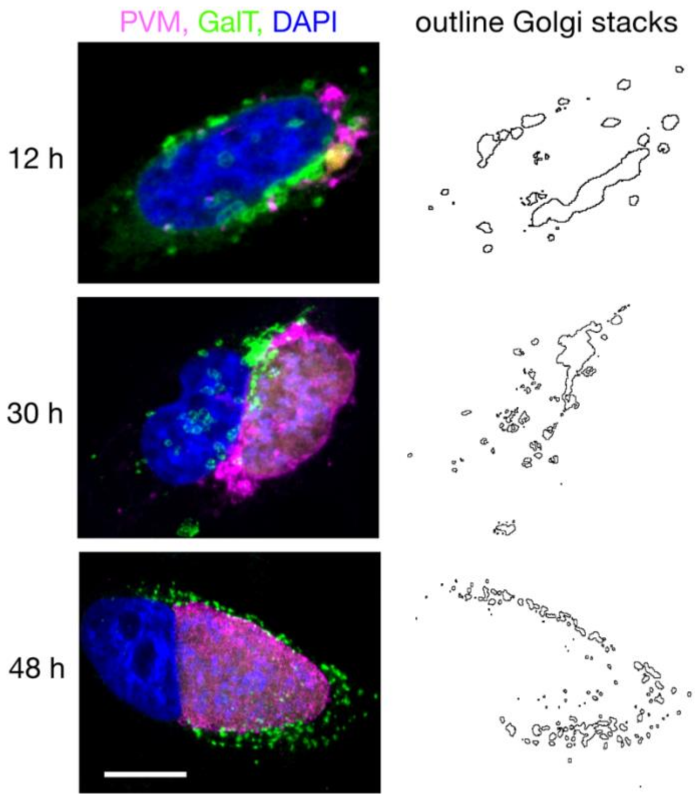

Figure S3. Representation of the automated segmentation of hcGolgi elements. Infected HeLa cells transiently expressing GalT-GFP (left panel) were fixed at time. The parasite's PVM stained for UIS4 to visualize the PVM (magenta) and DAPI (blue). **Left panel:** Immunofluorescence images of three representative time points during infection (12 hpi, 30 hpi and 48 hpi) highlight the progressing hcGolgi fragmentation. **Right panel:** displays the bare outlines of the Golgi structure of images on the left. Golgi outlines were obtained using Fiji to schematically show stack distribution, number, and area covered. Scale bars, 10 µm.

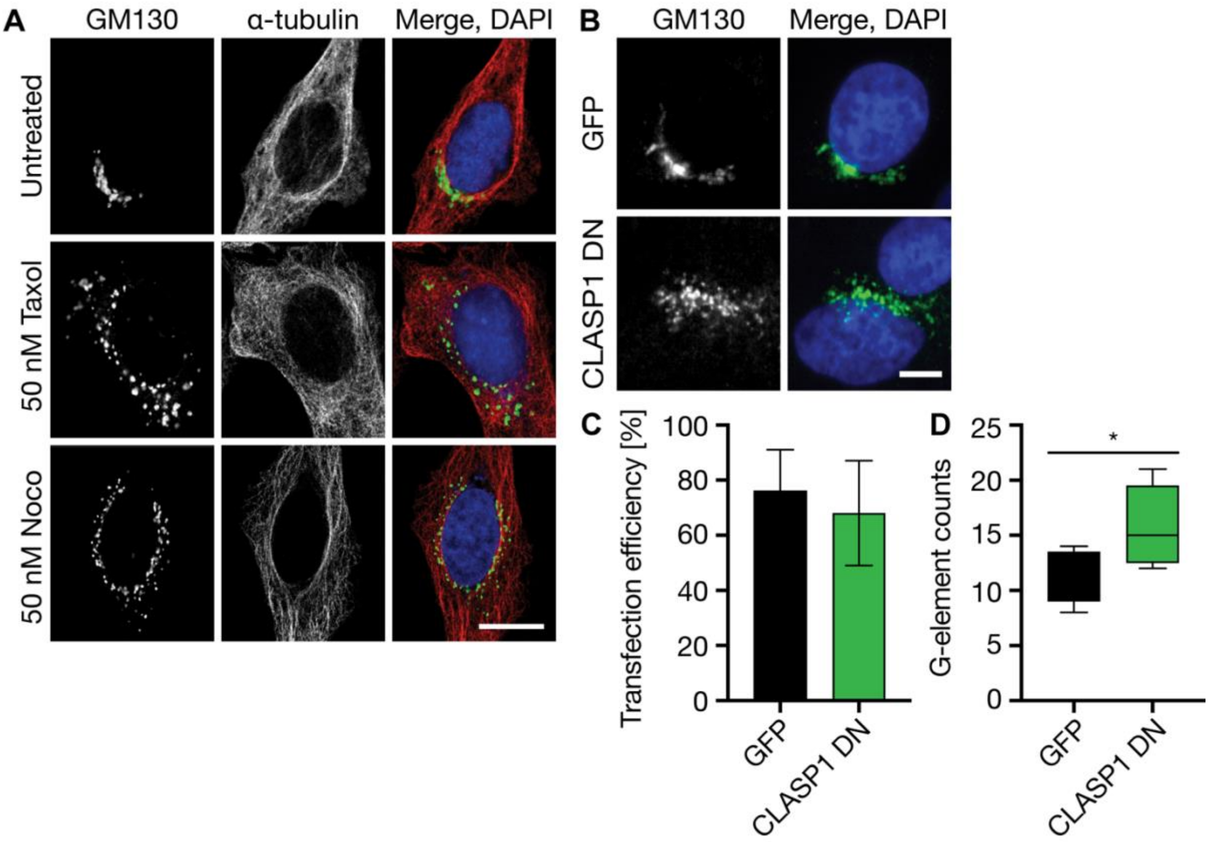

Figure S4. Manipulation of host cell microtubules result in a dismantled hcGolgi. A) HeLa cells treated with either 50 nM of Taxol or Nocodazole show changes in the organization of the MT and hcGolgi disassembly and scattering across host cell cytoplasm. Confocal images were taken from cells stained against the *cis*-Golgi protein GM130 (green), α-tubulin (red) and DAPI (blue). **B-D)** HeLa cell transiently overexpressing the microtubule binding domain of the CLIP-associating protein 1 (CLASP1) show loss of the Golgi architecture. hcGolgi of transfected cell was visualized with GM130 (green). **C)** Quantification of the transfection efficiency 12 h post transfection. Data were compiled from n = 3 experimental repeats. No significant difference was observed between the GFP control and CLASP1-DN. **D)** Quantification of the hcGolgi fragmentation caused by ectopical expression of CLASP1-DN in uninfected HeLa cells 24 h post transfection. The degree of hcGolgi fragmentation was analysed by counting the number of G-elements/cell. n = 3 independent experiments, * $p \leq 0.05$, one-way ANOVA. Scale bar, 10 µm.

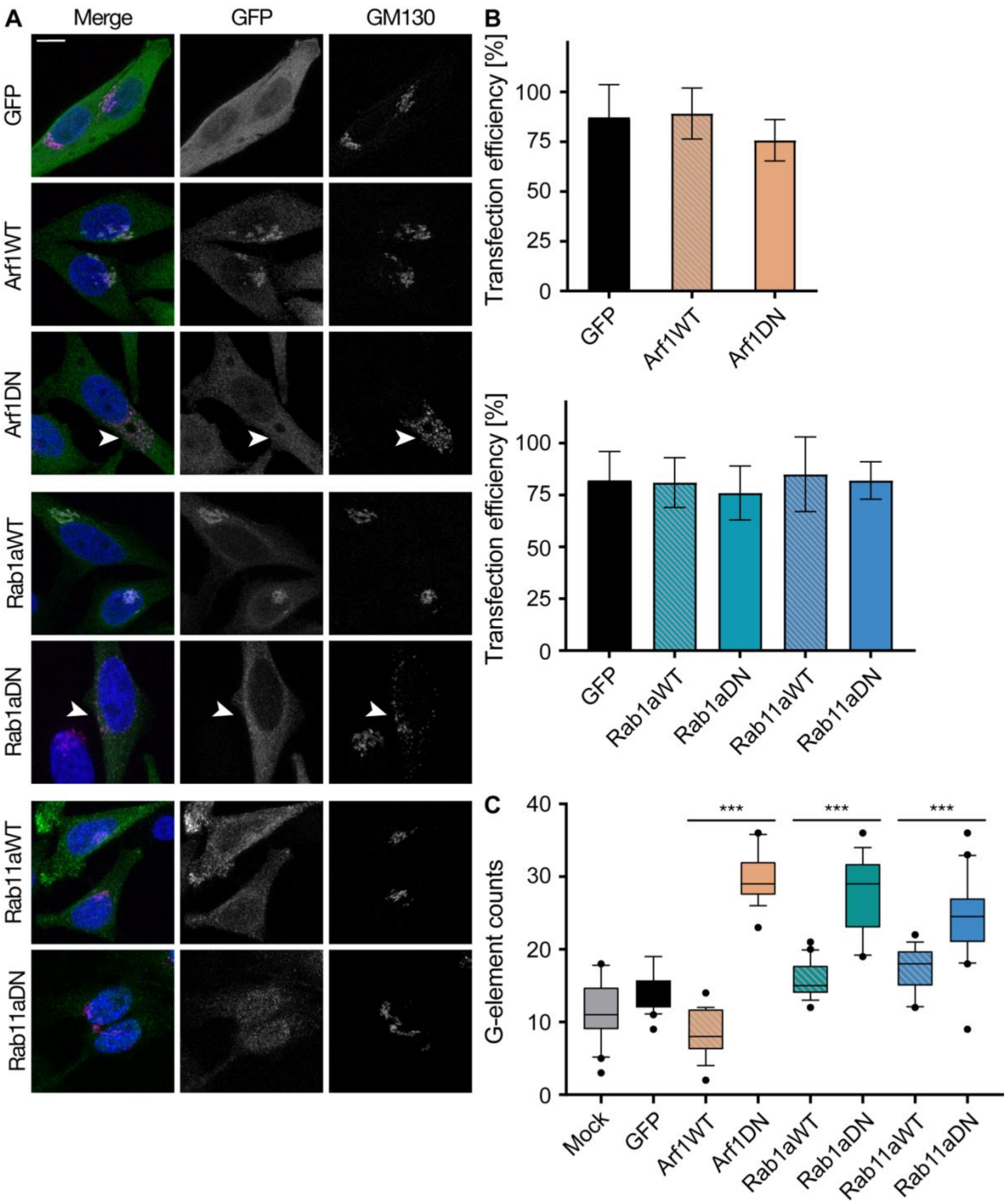

Figure S5. Analysis of the hcGolgi fragmentation phenotype induced by overexpression of regulators of the Golgi-associated vesicular transport. HeLa cells were transiently transfected with either a dominant negative (DN) or wild type (WT) version of Arf1, Rab1a and Rab11a. To assess a potential negative impact of the transfection on hcGolgi architecture, fragmentation was also analysed in mock and GFP transfected cells. **A)** Representative images (3D projections) of HeLa cells transiently overexpressing a dominant negative version of the small GTPases Arf1, Rab1a or Rab11a and the respective WT version or GFP. The effect of the respective small GTPase mutant on the hcGolgi fragmentation was assessed by staining for the *cis*-Golgi with α-GM130. White arrow head highlight the dispersal of *cis*-Golgi in Arf1 DN and Rab1a DN expressing HeLa cells. Images were acquired by confocal laser scanning microscopy (3D-CLSM) with 0.3 µm z-increments. Scale bar, 10 µm **B)** Quantification of the transfection efficiency 12 h post transfection. Data were compiled from all

Journal of Cell Science • Supplementary information

experimental repeats $n \geq 3$. No significant differences were observed between the constructs. **C)** Quantification of the hcGolgi fragmentation caused by ectopical expression of the dominant-negative Arf1, Rab1a and Rab11a in uninfected HeLa cells 24 h post transfection. The degree of hcGolgi fragmentation was analysed by counting the number of G-elements/cell. As a control served the mock transfected cells, cytosolic GFP and the respective WT. *** $p \leq 0.001$, one-way ANOVA.

Journal of Cell Science • Supplementary information

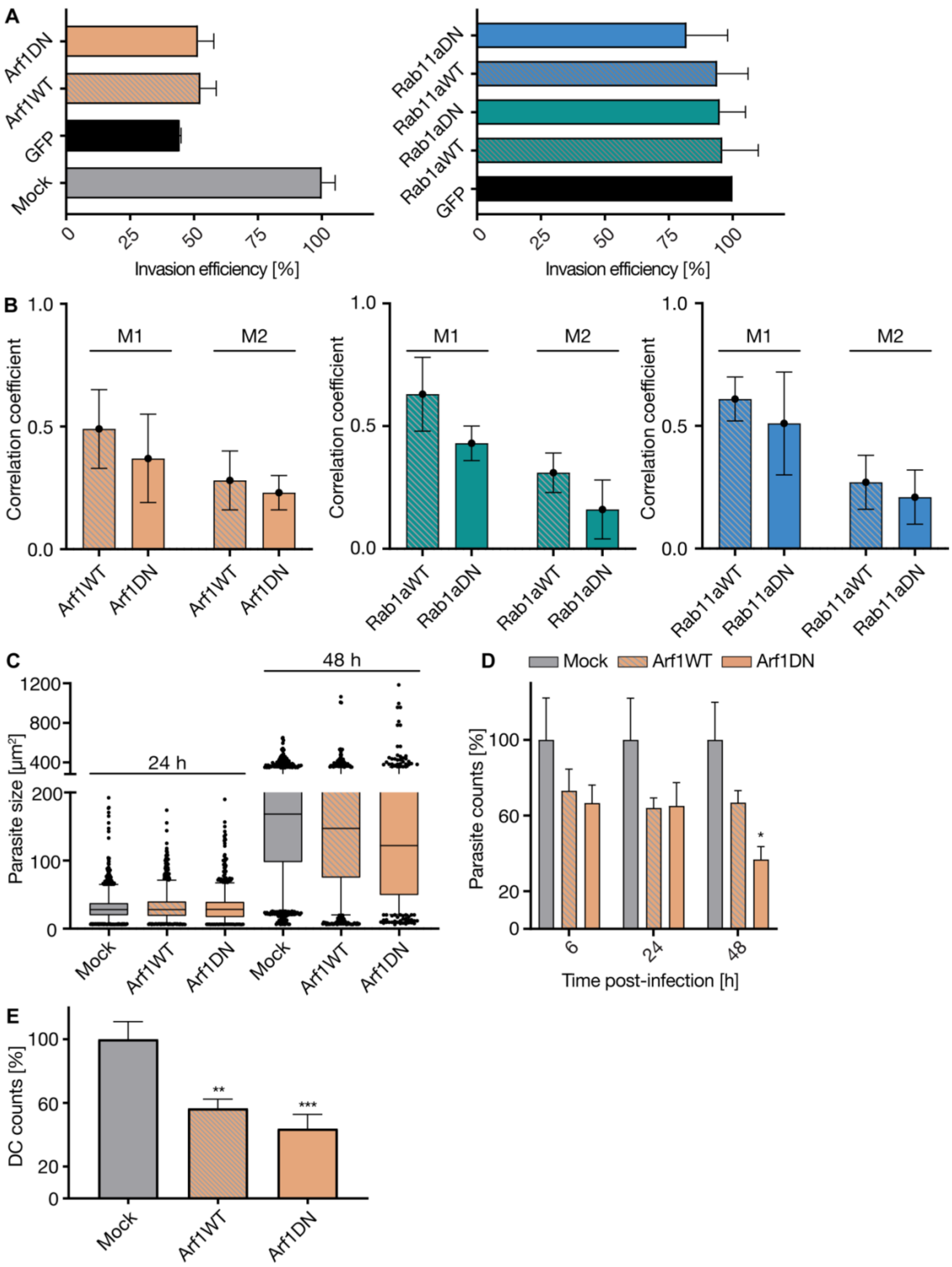

Figure S6. Quantification of parasite fitness and hcGolgi accumulation in host cells impaired in Golgi-associated vesicle transport. A) Parasite invasion efficiency was determined in HeLa cells transiently expressing the DN and WT version of Arf1, Rab1a and Rab11a. Cells were fixed at 2 hpi and stained with anti-GFP. Only infected cells expressing the construct, based on the detected GFP signal, were considered for the parasite invasion efficiency. To assess a potential negative impact of the transfection on parasite fitness, invasion efficiency of the parasite was also analysed in mock and

Journal of Cell Science • Supplementary information

GFP transfected cells. The difference of the invasion efficiency compared to the mock control is in consistence with the transfection efficiency (Fig. S5B). **B)** Alterations of PVM-hcGolgi interaction caused by the transient overexpression of DN of either Arf1, Rab1a or Rab11a was analysed young schizonts (24 hpi). Mander's overlap coefficient (MOC) was applied on the entire cell to determine the degree of co-localization between PVM and hcGolgi. In comparison to the respective WT GTPase no significant change in the distribution of the fluorescence signal was observed at 24 hpi. M1: portion of PVM overlapping with hcGolgi and M2: portion of hcGolgi overlapping with PVM. **(C-E)** High-content image-based characterisation of the parasite development in Arf1-deficient host cells. Parasite development in HeLa cells transiently overexpressing Arf1 DN or the respective WT were compared to mock transfected HeLa cells. **C-D)** Parasite size and numbers in transfected cells were quantified based on the detected GFP signal. **C)** Parasite growth was assessed based on parasite sizes at 24 and 48 hpi. **D)** Quantification of total parasite numbers during the first 48 h of infection (6, 24 and 48 hpi). Numbers are shown as a percentage of mock transfected (100%) at each time point. **E)** Percent detached cells (DCs) relative to the mock transfected control formed at 65 hpi. The total number of DCs was normalized to the number of parasites counted in the entire host cell population at 48 hpi. Therefore, number of DCs and 48 hpi schizonts also include parasites from untransfected cells. The data were compiled from 3 independent infections with $n > 200$ parasites, * $p \leq 0.05$, ** $p \leq 0.01$, *** $p \leq 0.005$, one-way ANOVA.

Journal of Cell Science • Supplementary information

Table S1. Golgi proteins studied in the context of Plasmodium exo-erythrocytic infections.

ER Golgi protein	Location	Function	Alteration by pathogens	References
**GalT**	*trans*-Golgi network	Membrane spanning Golgi enzyme. The glycosyltransferase catalyses the transfer of activated UDP-galactose on to an acceptor sugar.	Unknown	
GM130	*cis*-Golgi	Peripheral membrane protein at the Golgi. As vesicles tethering factor facilitates vesicle fusion to the Golgi membrane. Maintenance of the Golgi structure; major role in the disassembly and reassembly of the Golgi complex during mitosis. Involved in the control of glycosylation, cell cycle progression, and higher order cell functions such as cell polarization and directed cell migration.	Unknown	
**Rab proteins**				
Rab1	*cis*-Golgi	Regulates membrane-tethering events on three different pathways: autophagy, ER-Golgi traffic, and intra-Golgi traffic.	*Legionella pneumophila* targets Rab1 proteins. Their mechanism of action alters the destination of pathogen-occupied vacuoles to avoid fusion with lysosomes for destruction. Manipulation of Rab1 allows *L. pneumophila* to acquire nutrients that support bacterial propagation. *C. pneumonia* has been reported to interact with Rab1.	Sherwood and Roy, 2013; Stein et al., 2012
Rab2	*cis*-Golgi	Connects the Golgi network to autophagy pathway by delivering membrane and by sequentially engaging distinct autophagy machineries. Rab2 co-localizes with GM130 and regulates the formation and transport of COPI coated vesicles from the Golgi to the ER.	*Brucella sp.* recruits Rab2 to the *Brucella*-containing vacuole. Rab2 is thought to aid the VirB system for creating a host ER-derived vacuole that supports bacterial replication.	de Barsy et al., 2011; Sherwood and Roy, 2013
Rab6	*trans*-Golgi	Regulates protein transport from the Golgi complex to the ER and exocytosis along with the microtubules.	Rab6 recruited specifically to inclusions containing *Chlamydia trachomatis*. Rab6a, Rab11a and Rab14 are key for *C. trachomatis* development, by facilitating the transport of sphingolipids and cholesterol from vesicles shuttling between the Golgi and other organelles. Depletion of Rab6 or Rab11 decreased the formation of infectious particles.	Capmany and Damiani, 2010; Rejman Lipinski et al., 2009; Sherwood and Roy, 2013
Rab11	*trans*-Golgi and endosomes	Associates with perinuclear recycling endosomes and regulates recycling of endocytosed proteins. Causes tubulation of recycling endosomes, and gives rise to accumulation of recycling carriers containing endocytosed transferring and transferrin receptor beneath the plasma membrane.	*Chlamydia pneumoniae* and *Chlamydia trachomatis* manipulate Rab11 during infection. Important for *Legionella pneumophila* proliferation.	Sherwood and Roy, 2013
**Arf proteins**				
Arf 1	*cis*- and *trans*-Golgi network	Recruits COPI coat to membranes of the early secretory pathway. Recycles the trafficking machinery and escaped ER-resident proteins back to the ER. Recruits the Golgins to Golgi membranes. At contact sites between the ER and the Golgi, Arf1 is required for the recruitment of several lipid transfer proteins that mediate the transfer of sphingolipid precursors and cholesterol. Key role in establishing the lipid environment of cell membranes. Functions in lipid droplet metabolism.	Exploited by *Salmonella enterica* to remodel the actin cytoskeleton and invade host cells. *L. pneumophila* has been reported to recruit Arf1 to the *Legionella*-containing vacuole.	Brumell and Scidmore, 2007; Donaldson et al., 2005; Donaldson and Honda, 2005

Journal of Cell Science • Supplementary information

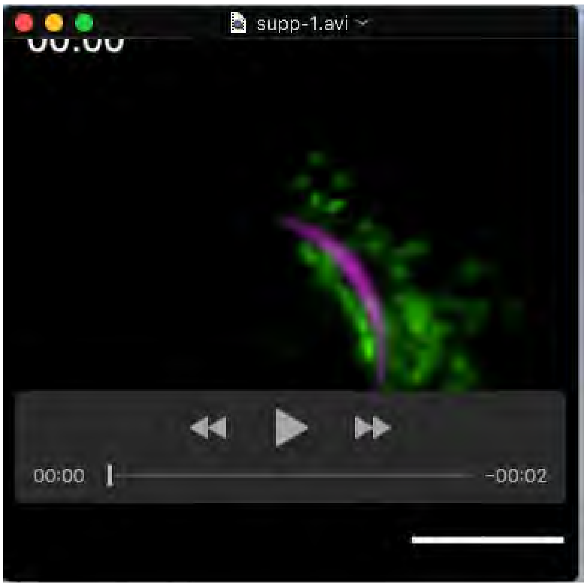

Movie 1. Invading sporozoites associates with hcGolgi. Huh7 cells transfected with the *trans*-Golgi enzyme GalT-GFP were infected with PbmCherry sporozoites and imaged by wide field time-lapse microscopy. The invaded sporozoite settles adjacent to *trans*-Golgi. Images were acquired every 5 min for the duration of 2 h. Time stamp, h:min, Scale bar, 10 µm

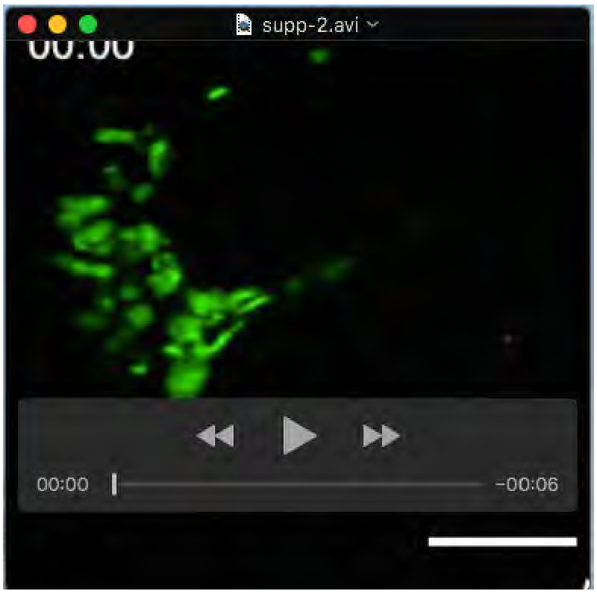

Movie 2. The parasite PVM and TVN interacts with *trans*-Golgi. A UIS4-mCherry trophozoite (7 hpi) was imaged in an GalT-GFP expressing Huh7 cells by confocal time-lapse microscopy. Dynamic interplay of the UIS4-positive TVN and PVM with the *trans*-Golgi of the host cell. Images were acquired every 30 s for the duration of 2 h. Time stamp, min:s, Scale bar, 10 µm

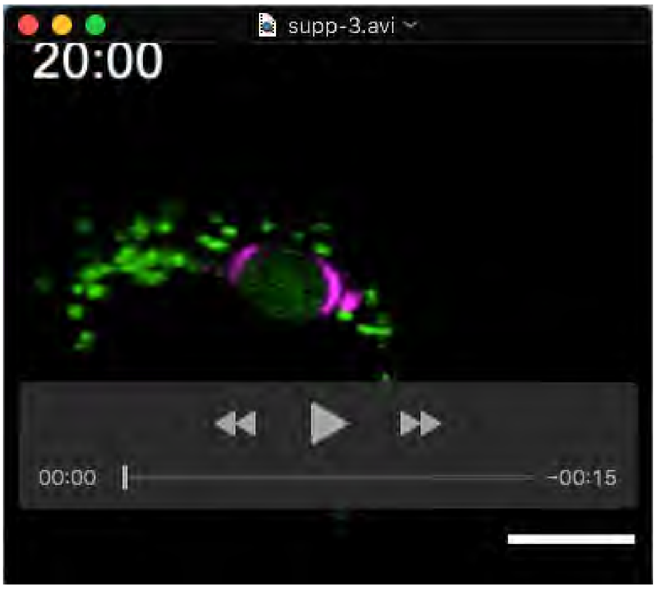

Movie 3. Parasite-induce hcGolgi fragmentation. A UIS4-mCherry young schizont (20 hpi) was imaged by confocal time-lapse microscopy in GalT-GFP expressing Huh7 cells. Over the time of infection the *trans*-Golgi fragments and loses its perinuclear location. Multiple hcGolgi elements (G-elements) interact with the UIS4-positive TVN and PVM likewise. Images were acquired every 5 min for the duration of 12 h. Time stamp, h:min, Scale bar, 10 µm

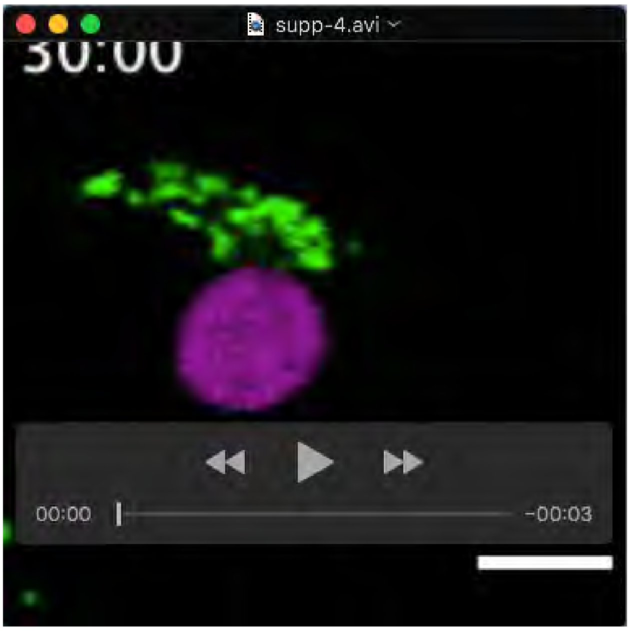

Movie 4. Parasite-induce hcGolgi fragmentation and redistribution. A mCherry young schizont (30 hpi) was imaged by confocal time-lapse microscopy in a Huh7 cells transiently transfected with the *trans*-Golgi marker GalT-GFP. The condense Golgi complex dissociates and redistributes around the growing schizonts. Images were acquired every 15 min for the duration of 7 h. Time stamp, h:min, Scale bar, 10 µm

Journal of Cell Science • Supplementary information
